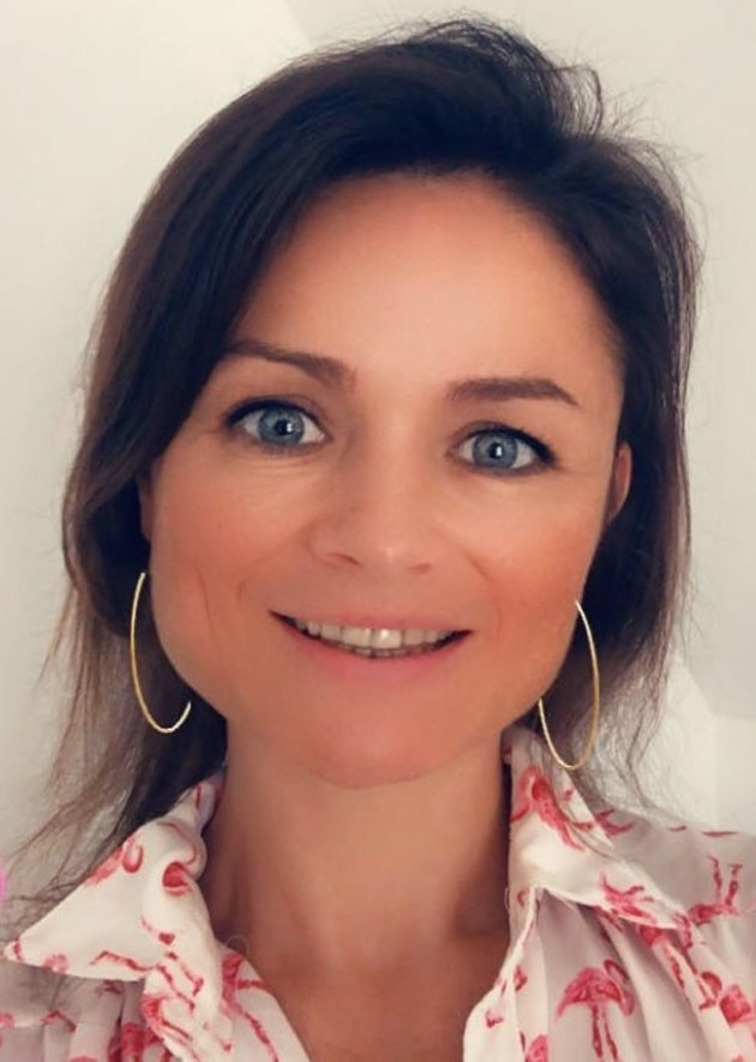# Strategies to Reduce the On‐Target Platelet Toxicity of Bcl‐x_L_ Inhibitors: PROTACs, SNIPERs and Prodrug‐Based Approaches

**DOI:** 10.1002/cbic.202100689

**Published:** 2022-03-19

**Authors:** Arvind Negi, Anne Sophie Voisin‐Chiret

**Affiliations:** ^1^ Department of Bioproduct and Biosystems Aalto University FI-00076 Espoo Finland; ^2^ Normandie University UNICAEN, CERMN 14000 Caen France

**Keywords:** Bcl-x_L_, Bcl-x_L_ inhibitors, drug conjugates, platelet toxicity, PROTACs, on-target toxicity, SNIPERs

## Abstract

Apoptosis is a highly regulated cellular process. Aberration in apoptosis is a common characteristic of various disorders. Therefore, proteins involved in apoptosis are prime targets in multiple therapies. Bcl‐x_L_ is an antiapoptotic protein. Compared to other antiapoptotic proteins, the expression of Bcl‐x_L_ is common in solid tumors and, to an extent, in some leukemias and lymphomas. The overexpression of Bcl‐x_L_ is also linked to survival and chemoresistance in cancer and senescent cells. Therefore, Bcl‐x_L_ is a promising anticancer and senolytic target. Various nanomolar range Bcl‐x_L_ inhibitors have been developed. **ABT‐263** was successfully identified as a Bcl‐x_L_/Bcl‐2 dual inhibitor. But it failed in the clinical trial (phase‐II) because of its on‐target platelet toxicity, which also implies an essential role of Bcl‐x_L_ protein in the survival of human platelets. Classical Bcl‐x_L_ inhibitor designs utilize occupancy‐driven pharmacology with typical shortcomings (such as dose‐dependent off‐target and on‐target platelet toxicities). Hence, event‐driven pharmacology‐based approaches, such as *pr*oteolysis *ta*rgeting *c*himeras (PROTACs) and SNIPERs (*s*pecific *n*on‐genetic *I*AP‐based *p*rotein *er*asers) have been developed. The development of Bcl‐x_L_ based PROTACs was expected, as 600 E3‐ligases are available in humans, while some (such as cereblon (CRBN), von Hippel‐Lindau (VHL)) are relatively less expressed in platelets. Therefore, E3 ligase ligand‐based Bcl‐x_L_ PROTACs (CRBN: XZ424, XZ739; VHL: DT2216, PZ703b, 753b) showed a significant improvement in platelet therapeutic index than their parent molecules (**ABT‐263**: DT2216, PZ703b, 753b, XZ739, PZ15227; **A1155463**: XZ424). Other than their distinctive pharmacology, PROTACs are molecularly large, which limits their cell permeability and plays a role in improving their cell selectivity. We also discuss prodrug‐based approaches, such as antibody‐drug conjugates (**ABBV‐155**), phosphate prodrugs (**APG‐1252**), dendrimer conjugate (**AZD0466**), and glycosylated conjugates (**Nav‐Gal**). Studies of *in‐vitro*, *in‐vivo*, structure‐activity relationships, biophysical characterization, and status of preclinical/clinical inhibitors derived from these strategies are also discussed in the review.

## Graphical abstract

**Bcl‐x_L_ protein expression** is essential for the survival of platelets, cancer, and senescence cells. Therefore, early‐stage anticancer/senolytic Bcl‐x_L_ inhibitor designs are plagued with on‐target platelet toxicity. Initial attempts with synergistic combinations allowed researchers to reduce effective Bcl‐x_L_ inhibitor dosage and related dose‐dependent toxicity. As these designs presented occupancy‐driven pharmacology, approaches based on event‐driven pharmacology (especially protein degradation approaches: PROTACs & SNIPERs) gained significant interest. Also, some prodrug strategies have been developed, which are discussed in detail in this review.

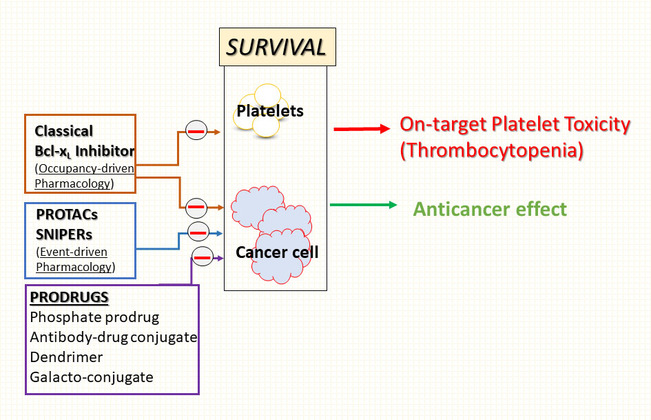

## 1 Introduction

**Figure 1:**
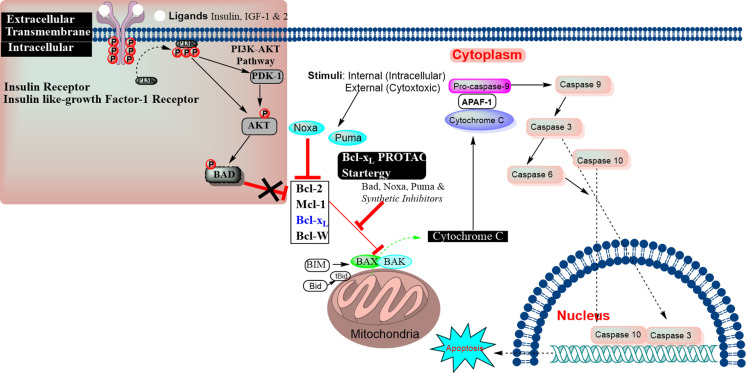
Apoptosis‐inducing proteins catalyze the MOMP process to release the cytochrome‐c. The cytochrome‐c forms an apoptosome complex with adaptor Apaf‐1 and caspase‐9 in the cytosol, and ultimately activates the caspase‐9 to initiate caspase‐cascade cellular apoptosis. The antiapoptotic proteins (Bcl‐2, Mcl‐1, and Bcl‐x_L_) inhibit the cytochrome‐c release, whereas Bax, Bak, Bid and other apoptosis‐inducing proteins, promote the release of cytochrome‐c from mitochondria. The inhibitors of Bcl‐2/Mcl‐1/Bcl‐x_L_ prevent PPIs of Bcl‐2/Mcl‐1/Bcl‐x_L_ with apoptosis‐inducing proteins (BH3 helix of Bid, Bim, Bad, Puma, Bmf, and Noxa), which increase the cellular level of BAX/BAK, their availability for oligomerization, which eventually leads to the release of cytochrome‐c.[^1^, ^2^] The Insulin‐like growth factor receptor and insulin receptor tyrosine kinase initiate the phosphorylation which activates the intracellular accessary signaling cascade *via* the PI3 K‐AKT pathway,^[3]^ resulting in activating the BAD into its phosphorylated form, which then modifies the mitochondrial functions in senescent cells.^[4]^

Apoptosis is a highly regulated mitochondrial process. It plays a critical role in remodeling, aging, tumorigenesis, and other cellular processes. Any alteration in the apoptosis leads to various cellular states, such as malignancy, neurodegeneration, and accumulation of senescent cells in age‐related diseases. The protein‐protein interactions (PPIs) of Bcl‐2 family members regulate cellular apoptosis by altering the mitochondrial outer membrane permeabilization (MOMP). Based on the physiological role of members of the Bcl‐2 protein family, there are two classes: *Antiapoptotic proteins* (Bcl‐x_L_, Mcl‐1, Bcl‐W, and Bfl‐1/A1), which inhibit MOMP formation; *Apoptosis‐inducing proteins*, which divide into two subtypes based on their structural differences: one‐subtype called as, “BH3‐only proteins” (Bik, Bim, Bid, Bad, Bmf, Hrk, Noxa, and Puma), while second‐subtype comprise protein members (such as Bax and Bak proteins) oligomerize to form a pore in the mitochondrial membrane (as shown in Figure 1).

Therefore, any dysregulation in the apoptosis pathway severely affects the survivability and longevity of the cell. The antiapoptotic proteins (Bcl‐2, Mcl‐1,^[1]^ and Bcl‐x_L_) are the major disrupters of cellular apoptosis signaling. Various studies reported a higher expression of Bcl‐x_L_ in solid tumors, a subset of leukemia, and lymphomas than other antiapoptotic members.^[5]^ Also, some studies found a strong correlation between the overexpression of Bcl‐x_L_ protein with acquiring resistance to other anticancer drugs.^[6]^ Therefore, Bcl‐x_L_ is one of the most promising cancer targets. As expected, the higher expression of Bcl‐x_L_ in solid tumors led to a clinical investigation of a dual Bcl‐x_L_/Bcl‐2 inhibitor (**ABT‐263**, also called **navitoclax**), which exhibited frequent dose‐dependent thrombocytopenia. The occurrence of **ABT‐263** induced thrombocytopenia in clinical trials implied that the expression of Bcl‐x_L_ is crucial for the survival of the platelets. Therefore, to reverse the sensitivity of Bcl‐x_L_ dependent‐resistant cancer cells or to attain cell‐selectivity towards Bcl‐x_L_ over‐expressed senescent/cancer cells requires a Bcl‐x_L_ inhibitor devoid of platelet toxicity.

## 2 Clinical Issues of Bcl‐x_L_ Inhibitors

**Figure 2:**
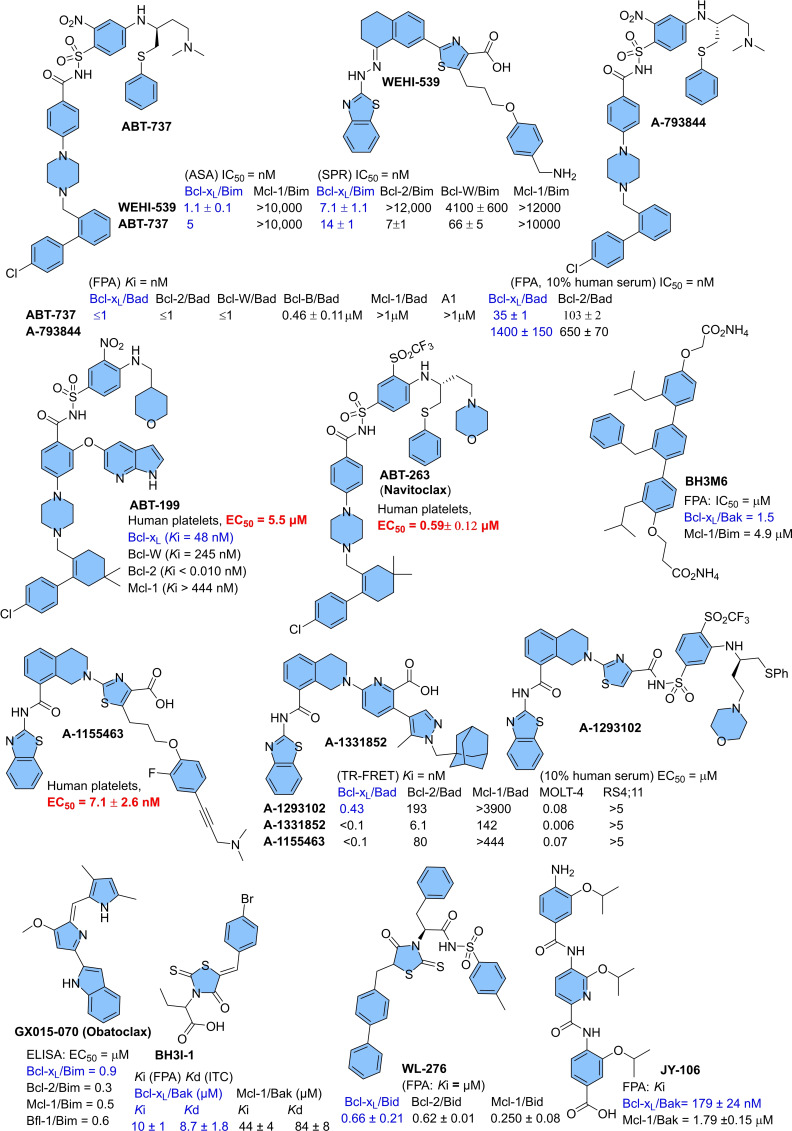
Synthetically developed inhibitors of Bcl‐x_L_ protein with associated platelet toxicities: **ABT‐199**,^[13]^ **ABT‐263**,^[13]^ **BH3I**‐**1**,^[14]^ **WL‐276**,^[15]^ **Obatoclax**,^[16]^ **WEHI‐539**,^[17]^ **A‐793844**,^[7]^ **ABT‐737**,[^7^, ^17^] **A‐1293102**,^[18]^ **A‐1331852**,^[18]^ **A‐1155463**,^[18]^; Oligomers: *p*‐terphenyl derivative (**BH3‐M6**),[^17^, ^19^] oligoamide‐foldamer (**JY‐1**‐**106** (IC_50_: Bcl‐x_L_/Bak=394±54 nM,^[20]^ Mcl‐1/Bak=10.21±0.83 μM^[21]^)). ELISA: enzyme‐linked immunosorbent assay.

**Figure 3:**
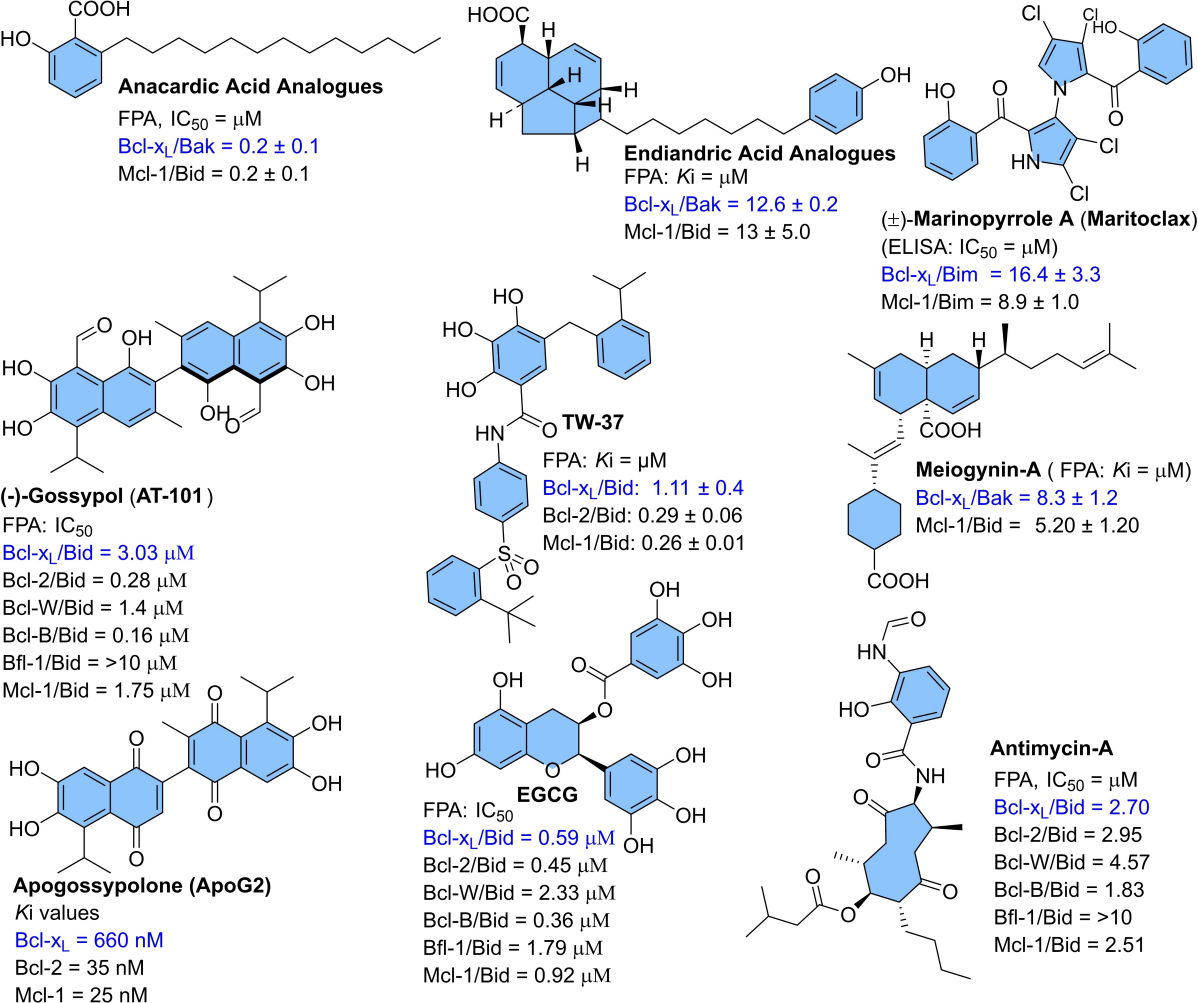
Naturally derived preferential or dual Bcl‐x_L_/Bcl‐2/Mcl‐1 inhibitors: Anacardic acids,^[22]^ Endiandric acids,^[23]^ Marinopyrroles,^[24]^ Polyphenols (Gossypol, ApoG2, EGCG, TW‐37),[^25^, ^26^] Meiogynins,^[27]^ and antimycin‐A.^[26]^ However, most of them were not studied for their on‐target platelet toxicity.

Various synthetic and naturally derived Bcl‐x_L_ inhibitors were identified (as shown in Figures 2 and 3). **ABT‐737** was the first potent dual inhibitor (Bcl‐x_L_/Bcl‐2) derived from the NMR‐guided fragment‐based drug design,[^7^, ^8^] which later optimized to an orally active analog (**ABT‐263**).^[9]^ During phase‐1 dose‐escalation study in lymphoid malignancies (*clinical trial number*. NCT00406809), **ABT‐263** showed a high therapeutic index along with off‐target toxicities, including low‐grade gastrointestinal disorders. However, it also showed a pharmacodynamic effect on circulating lymphocytes and platelets. Currently, an evaluation of the safety profile of **ABT‐263** for small cell lung cancer (SCLC) is in Phase II clinical trials. Other well‐studied inhibitors, such as **gossypol** and **obatoclax**, were also found with no approved clinical indications and only used as research tools. The clinical studies of **gossypol** showed a dose‐dependent gastrointestinal disorder, while the phase‐1 study of mesylate salt of **obatoclax** exhibited the dose‐limiting neurological symptoms with occasional episodes of grade‐3/4 neutropenia and thrombocytopenia.^[10]^ Also, similar dose‐dependent toxicities were observed with other inhibitors: (**BM‐1197**,^[11]^ **ABT‐737**,^[7]^ and **A‐1155463**.^[12]^ However, **ABT‐199** (a selective Bcl‐2 inhibitor also named **venetoclax**, **RG7601**, **GDC‐0199**) is clinically approved for blood cancers (chronic lymphocytic leukemia CLL, small lymphocytic lymphoma SLL, and acute myeloid leukemia AML), but not for solid tumors. To understand such pharmacology differences of **ABT‐199**, further studies conducted on solid tumors revealed their survival not only depends on Bcl‐2 protein signaling but also on Bcl‐x_L_ protein signaling. The reported synthetic inhibitors (Figure 2) and naturally derived inhibitors (Figure 3) for Bcl‐x_L_ protein, found with off‐target activities and multiple protein binding affinities (Bcl‐2/Mcl‐1/Bcl‐x_L_) affinities, showing an intrinsic flaw in their designs.

**Figure 4:**
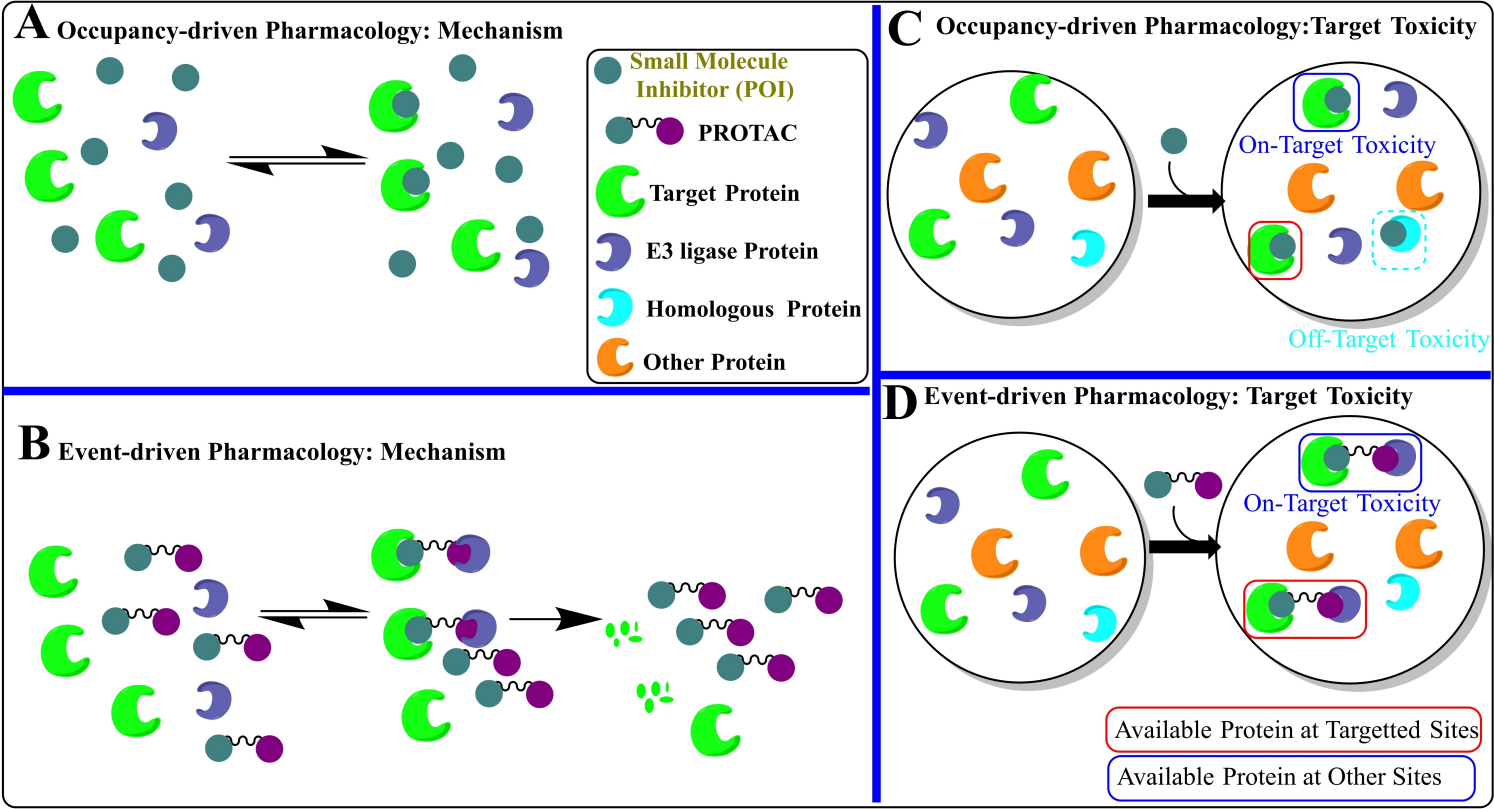
(A) Occupancy‐driven pharmacology target toxicity: This type of pharmacology is mainly shown by classical inhibitors (green color spheres) which inhibit or modify the signaling of a protein of interest (POI) (green crescent). However, the success of a complete inhibition is proportional to the time‐dependent bound concentration of SMI to the POI, therefore, these approaches require highly potent affinities of SMIs towards the POI. (B) Representation of event‐driven pharmacology: A bifunctional molecule (dumbbell‐shaped) subsequently binds to the target protein (green crescent) and E3 ligase protein (violet crescent). The binding with E3 ligase (violet crescent) induces the POI hydrolysis, *e. g*. protein degradation approaches. (C) Occupancy‐driven pharmacology target toxicity: Off‐target toxicity, as well as on‐target toxicity, are typical with SMIs as a virtue of their smaller sizes. Their smaller sizes and high potency, not only allow them to target the POI in other non‐relevant cells (on‐target toxicity) but also inherited them with a flaw to adopt non‐selective entropy binding conformation with homologous (orange crescent) as well as non‐homologous proteins (cyan crescent). (D) Occupancy‐driven pharmacology target toxicity: These strategies mainly use large structures, which reduces their cross‐membrane permeation and the degree of entropy‐based non‐selective conformations for homologous as well as non‐homologous proteins, and therefore relatively less prone to the off‐target toxicities. However, the wider cellular availability of target protein leads to minimal‐to‐moderate on‐target toxicity from these approaches.

As initial development of Bcl‐x_L_ inhibitors based on occupancy‐driven pharmacology (that means they block the PPIs of Bcl‐x_L_ with apoptosis‐inducing proteins) (as shown in Figure 4A), therefore plagued with typical limitations of such approach: (a) frequent occurrence of acquired resistance, (b) a continuous administration of higher dosage is required to maintain the therapeutic level, and (c) requirement of nanomolar‐to‐picomolar range inhibitors to achieve a full target inhibition. Therefore, inhibitors based on such pharmacology lack cell‐specificity and features to differentiate among proteins that exhibit high structural homology in their three‐dimensional structure.^[28]^ Therefore, on‐target platelet toxicity of Bcl‐x_L_ inhibitor is unlikely to be resolved with classical small molecular inhibitors (SMIs). Hence, event‐driven pharmacology inhibitor designs (as shown in Figure 4B) have gained interest in recent years. In comparison, the event‐driven pharmacology inhibitors degrade the target protein rather than blocking its interaction with a partner protein. Therefore, frequent dosing for continuous targeting is less required, and such inhibitors are relatively less prone to the emergence of resistance. Contrary to the occupancy‐driven pharmacology approaches, which require subnanomolar potency of inhibitors, event‐driven pharmacology approaches don't require molecules of subnanomolar affinities and, therefore, have a lesser tendency to produce dose‐dependent on‐target toxicity (as shown in Figure 4C and Figure 4D).

## 3 Strategies to Address the On‐Target Bcl‐x_L_ Platelet Toxicity

Various strategies have been implemented to reduce the on‐target platelet toxicity of Bcl‐x_L_ inhibitors. Most of the developed inhibitors belonged to the event‐driven pharmacology‐based approaches (PROTAC and SNIPER‐based design), with some examples of prodrug approaches (phosphate‐prodrugs, dendrimers, and glycosylation).^[29]^ Unlike the differences in the strategies, the pharmacodynamic objective remains the same: to reduce the platelet toxicity of Bcl‐x_L_ inhibitors by incorporating newer elements that can take advantage of those enzymes or proteins specifically expressed in cancer and senescent cells than platelets.

### 3.1 Synergistic combination of inhibitors

As expected, the initial efforts were attempted with the conventional approaches, where potent Bcl‐x_L_ inhibitors were used in combination with other clinical agents. The synergistic effect of such combinations allowed to reduce the dose of Bcl‐x_L_ inhibitors in those combinations, and their dose‐dependent platelet toxicity to an extent. The most notable examples are the combination of **ABT‐263** and **JQ‐1** (bromodomain inhibitor) that exhibited a synergistic role against *MYCN*‐amplified SCLC,^[30]^ and the combination of **ABT‐263** with **docetaxel**. However, the availability of clinical agents that can be used in combination with Bcl‐x_L_ inhibitors is one of the major limitations, and therefore other strategies were developed, such as (a) Bcl‐x_L_ targeted PROTACs, (b) Bcl‐x_L_‐targeted SNIPERS, (c) Prodrugs‐based Bcl‐x_L_ inhibitors.

### 3.2 Bcl‐x_L_ targeted PROTACs in cancer

**Figure 5:**
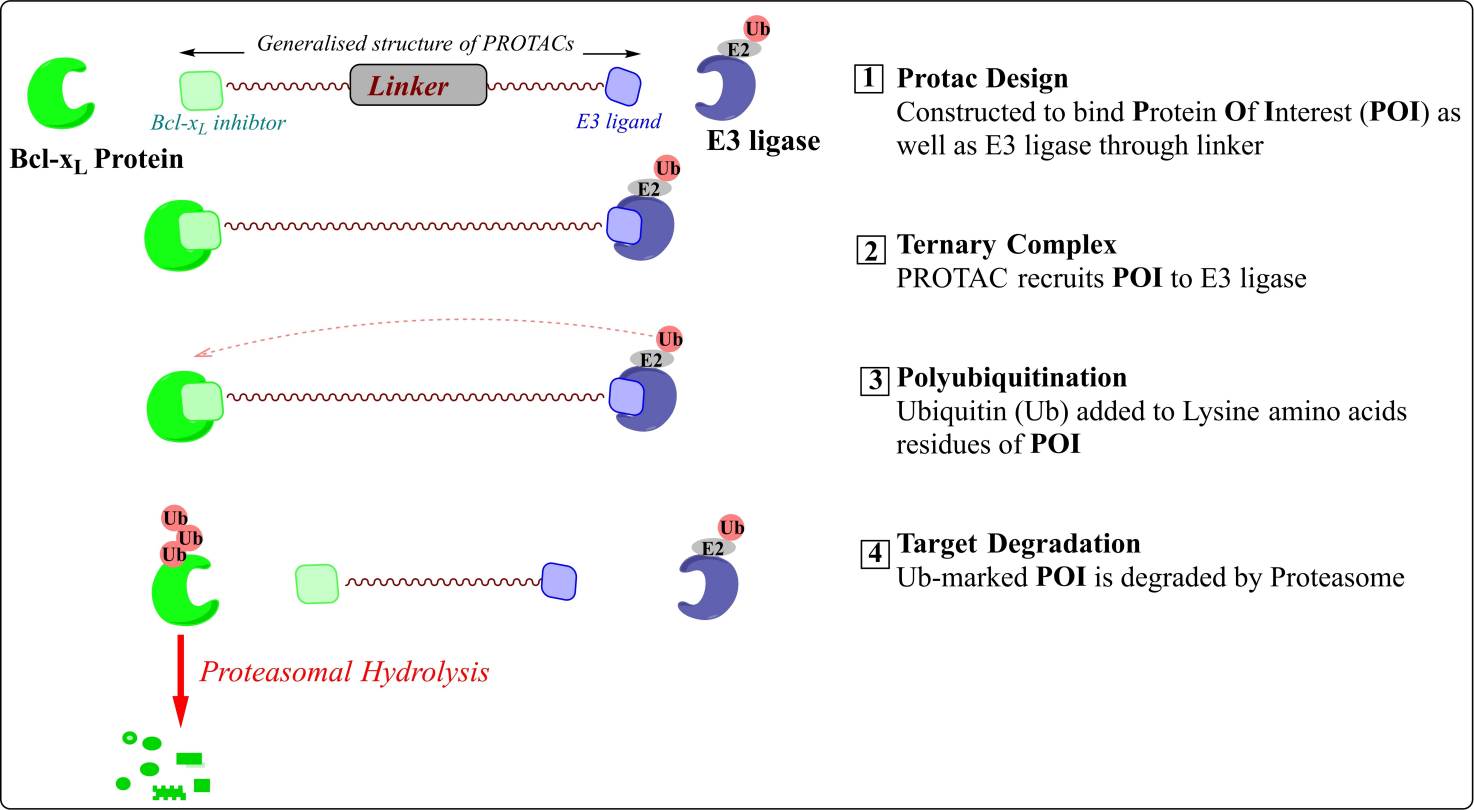
Schematic representation and mechanism involved in PROTACs targeted protein degradation. The PROTACs inhibition is usually measured in DC_50_ (half‐maximal degradation concentration) and D_max_ (maximum degradation).

The PROTACs (*PRO*teolysis *T*Argeting *C*himeras) are heterobifunctional medium‐sized molecules that promote selective and rapid proteosome‐mediated degradation of intracellular proteins via recruiting the E3 ligase complexes to the non‐native protein substrates. Chemically, these comprise two distinct pharmacophores (also called “warheads”), which are tethered together by a linker (as shown in Figure 5). The generalized structure for Bcl‐x_L_‐PROTAC contains a Bcl‐x_L_ pharmacophore at one terminal that helps it to bind to Bcl‐x_L_ protein while the other pharmacophore on the other side of PROTAC binds to E3 ligase. The subsequent binding of PROTAC to Bcl‐x_L_ and E3‐ligase brings E3 ligase in proximity to the Bcl‐x_L_ protein, which promotes the transfer of ubiquitin (Ub) units to the exposed lysine amino acids that are present on the surface of Bcl‐x_L_ protein. As shown in Figure 5, the presentation of Ub units on the surface of Bcl‐xL leads to its hydrolysis. Unlike conventional SMIs, the PROTAC molecule can be recovered from each protein hydrolysis and can repetitively take part in successive protein hydrolysis. Therefore, Bcl‐x_L_ PROTACs require hooking themselves with the Bcl‐x_L_ protein with moderate affinity, which is contrary to classical Bcl‐x_L_ SMIs as they require high affinity and, often suffer from dose‐dependent toxicities and a lower therapeutic index. The success of a PROTAC molecule is based on its ability to form a ternary complex (in this case, Bcl‐x_L_:PROTAC: E3‐Ligase). The formation of a ternary complex from a binary complex is an example of cooperative binding. It means that the first binding of either pharmacophore to its protein (in this case, Bcl‐x_L_ pharmacophore to Bcl‐x_L_ protein and E3 ligase ligand to E3‐ligase) influences the binding of another pharmacophore for its protein, which could lead to the formation of binary complexes rather than ternary complexes (a biophysical limitation, also termed as Hook's effect). To achieve positive cooperatively in PROTAC strategy, the researchers explored the length and chemical nature of the linker region of PROTAC, rather than derivatizing the pharmacophores as that would pose a high risk of loss in affinity towards their respective proteins. Hence, various linker designs have been presented with a wide PROTAC‐type implementation,^[31]^ such as amide‐to‐ester substitution,^[32]^ azide‐alkyne cycloaddition,^[31]^ and diazo reactions.^[33]^ Also, a focus has been drawn on the choice of E3 ligase ligands as the expression of E3 ligases is cell‐specific.^[34]^ Also, a lower level of the E3 ligase ((Von Hippel‐Landau (VHL),^[35]^ cereblon (CRBN)), associated E1 (UBA1), and E2 (SFT) enzymes found in human platelets.

**Figure 6:**
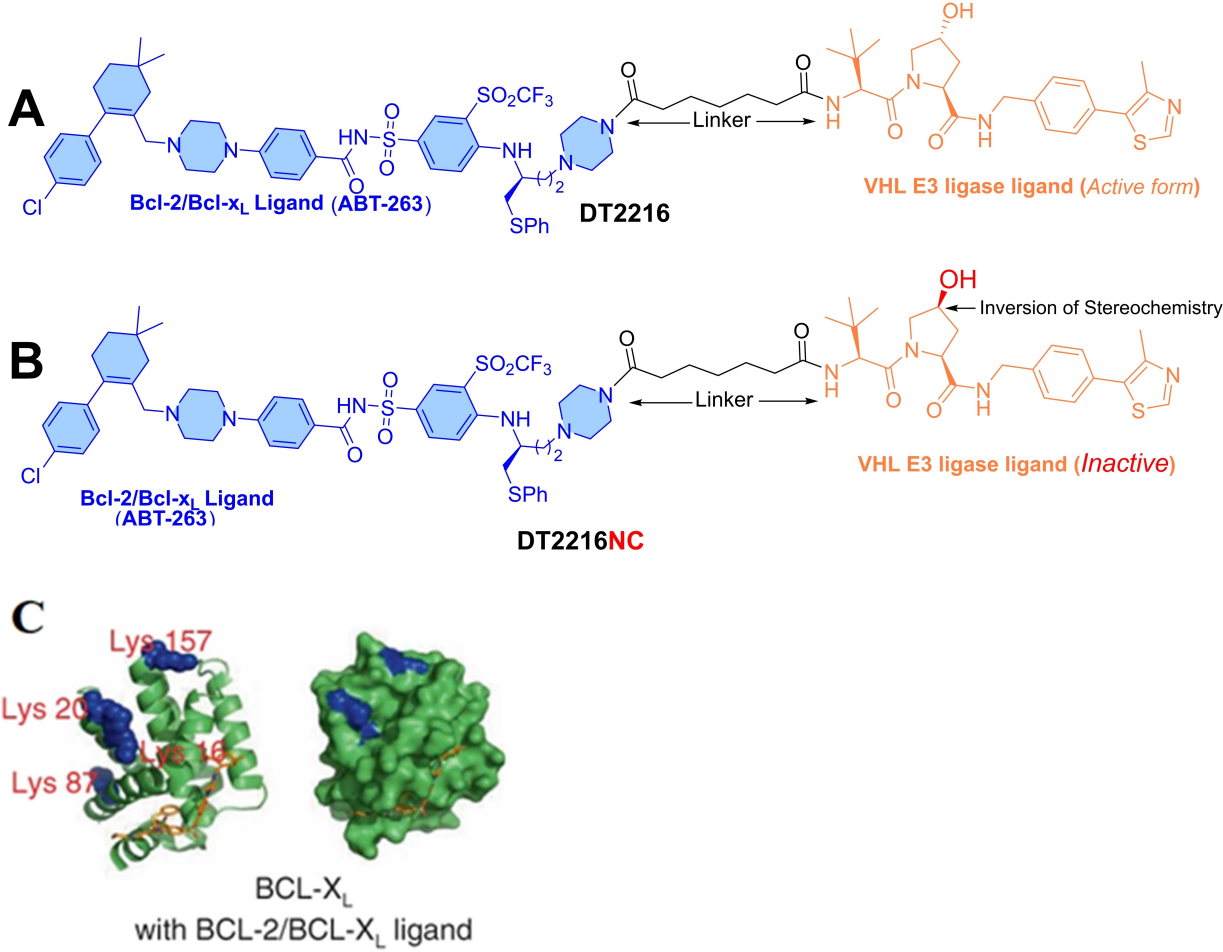
(A) Chemical structure of **DT2216** for Bcl‐x_L_ degradation containing **ABT‐263** (left‐hand side) and VHL E3 ligase ligand (right‐hand side). (B) Chemical structure of **DT2216NC** for Bcl‐x_L_ degradation containing **ABT‐263** (left‐hand side) and hydroxylated version of VHL E3 ligase ligand (right‐hand side), which doesn't bind to the VHL. (C) Showcasing the surface lysine residues in Bcl‐x_L_ (Left side) and Bcl‐2 (Right side), reproduced with permission from Khan et al.^[36]^ Copyright 2019 Nature Publishing Group.

This finding led the researchers to incorporate E3 ligase ligands into the Bcl‐x_L_ PROTAC strategy to reduce the on‐target platelet toxicity. In 2019, Zheng and co‐worker from the College of Pharmacy, University of Florida identified **DT2216** as the first example of Bcl‐x_L_ PROTAC, which has an **ABT‐263** as a Bcl‐x_L_ warhead and VHL‐recruiting E3‐ligase ligand (as shown in Figure 6A).^[36]^ The adapted synthetic strategy to develop **DT2216** includes a replacement of the morpholine ring of **ABT‐263** with piperazine. The piperazine ring served as an attachment point for the VHL‐ligase ligand through a 6‐carbon amide linker (as shown in Figure 6A).^[36]^ In MOLT‐4 cells (Bcl‐x_L_‐dependent T‐cell acute lymphoblastic leukemia, “T‐ALL”), the **DT2216** showed a rapid and long‐lasting (D_max=_90.8 %) Bcl‐x_L_ protein degradation (DC_50=_63 nM). However, moderate on‐target platelet toxicity of **DT2216** was also observed (D_max_=26 %) up to a 3 μM dose level. The viability assay on MOLT‐4 cells showed a four‐fold potency of **DT2216** (EC_50_=52 nM**)** over **ABT‐263** (EC_50_=191 nM), with no platelet cytotoxicity until 3 μM.^[36]^ Preincubation of MOLT‐4 cells with an excess of **ABT‐263**, **VHL**, and **MG132** (a proteasome inhibitor) showed a significant reduction in ternary complex (Bcl‐x_L_‐DT2216‐VHL) formation and Bcl‐x_L_ degradation. Also, no effect on the cellular level of Bcl‐x_L_ in VHL‐null 786‐O renal cell carcinoma cells was recorded. Later, negative control of **DT2216** (**DT2216‐NC**) was developed by inverting the stereochemistry on the hydroxyproline moiety of a VHL ligand (as shown in Figure 6B). This inverted stereochemistry doesn't allow the PROTAC molecule (**DT2216‐NC**) to adopt a binding conformation with E3 ligase, therefore as expected, **DT2216‐NC** failed to induce Bcl‐x_L_ degradation and, no ternary complex formation was observed. These observations supported the **DT2216**‐induced proteasome‐mediated Bcl‐x_L_ degradation. Later, various *in‐vivo* efficacy parameters of **DT2216** were evaluated in MOLT‐4, NCI−H146 (small cell lung carcinoma, SCLC), MDA‐MB‐231 (breast cancer) xenograft mouse models. In MOLT‐4 xenografts, a single dose of intraperitoneal injection (i. p. dose=15 mg/kg) of **DT2216** produced a significantly higher intra‐tumoral concentration than cellular EC_50_ values, that were retained for more than one week and led to a substantial reduction in the Bcl‐x_L_ expression. Also, a mild reduction in platelet count with no reactive thrombocytosis was observed after treatment of **DT2216**, whereas an equivalent therapeutic dose of **ABT‐263** produced severe thrombocytopenia after 6 h. Based on these outcomes, **DT2216** was found more efficacious and safer antitumor agent than **ABT‐263**. The significant improvement in platelet toxicity as shown by **DT2216** over **ABT‐263** exhibits a plausible role of changes in physicochemical properties during the transition of **DT2216** from **ABT‐263**. As the changes in physiochemical properties are directly affected by the presence of an extra‐large structure of **DT2216** (also called “*molecular obesity*”) than its parent **ABT‐263** (which has a smaller heterocyclic structure), which certainly attributed to lowering its cell permeability. Furthermore, the cytotoxicity profile of **DT2216** was compared with the clinical inhibitors of other Bcl‐2 family proteins as shown in Table 1.

**Table 1:** Comparison of sensitivity of promising Bcl‐2 family inhibitors with respect to **DT2216**.

Cancer	Cell	Sensitive: (EC_50_=μM)			EC_50_=μM	
type	line	A‐1155463 (Bcl‐x_L_)	ABT‐199 (Bcl‐2)	S63845 (Mcl‐1)	ABT‐263 (Bcl‐2/Bcl‐x_L_)	DT2216
T‐ALL	MOLT‐4	high	insensitive	insensitive	0.191	0.052
B‐ALL	RS4;11	insensitive	high	insensitive	0.028	0.23
multiple myeloma	EJM	insensitive	insensitive	moderate	>2	>2
	H929	insensitive	insensitive	high	>2	>2
SCLC	NCI−H146	high	moderate	NR	0.030	0.160
breast	MDA‐MB‐231	moderate	insensitive	insensitive	0.707	0.229
prostate	PC3	insensitive	insensitive	insensitive	>10	>10
hepatic	HepG2	insensitive	insensitive	insensitive	>10	>10
colon	SW620	insensitive	insensitive	insensitive	>10	>10
renal	786‐0	NR	NR	NR	>10	>10

NanoBRET assay measured the ternary complex formation ability of **DT2216** with Bcl‐x_L_ and Bcl‐2 protein in live cells, where a ternary complex was observed for Bcl‐x_L_ but not for Bcl‐2, though **DT2216** showed a higher Bcl‐2 protein binding affinity than Bcl‐x_L_.

To understand such behavior of **DT2216**, the co‐crystal structure of **ABT‐263** with Bcl‐x_L_ (PDB code: 4QNQ) was studied. It was observed that the solvent‐exposed lysine residues for potential ubiquitin sites are available at 16, 20, 87, and 157 (as shown in Figure 6C), with the other two lysine residues (205 and 233). As later lysine residues (205 and 233) are buried in the transmembrane region, therefore were not considered for further studies. Mutation analysis showed that lysine 87 is the key residue available for ubiquitination that induces Bcl‐x_L_ degradation. Whereas, the corresponding lysine residue is missing on the surface of Bcl‐2 protein, which also explained why **DT2216** was selectively Bcl‐x_L_ degrader but not a Bcl‐2 degrader.

**Figure 7:**
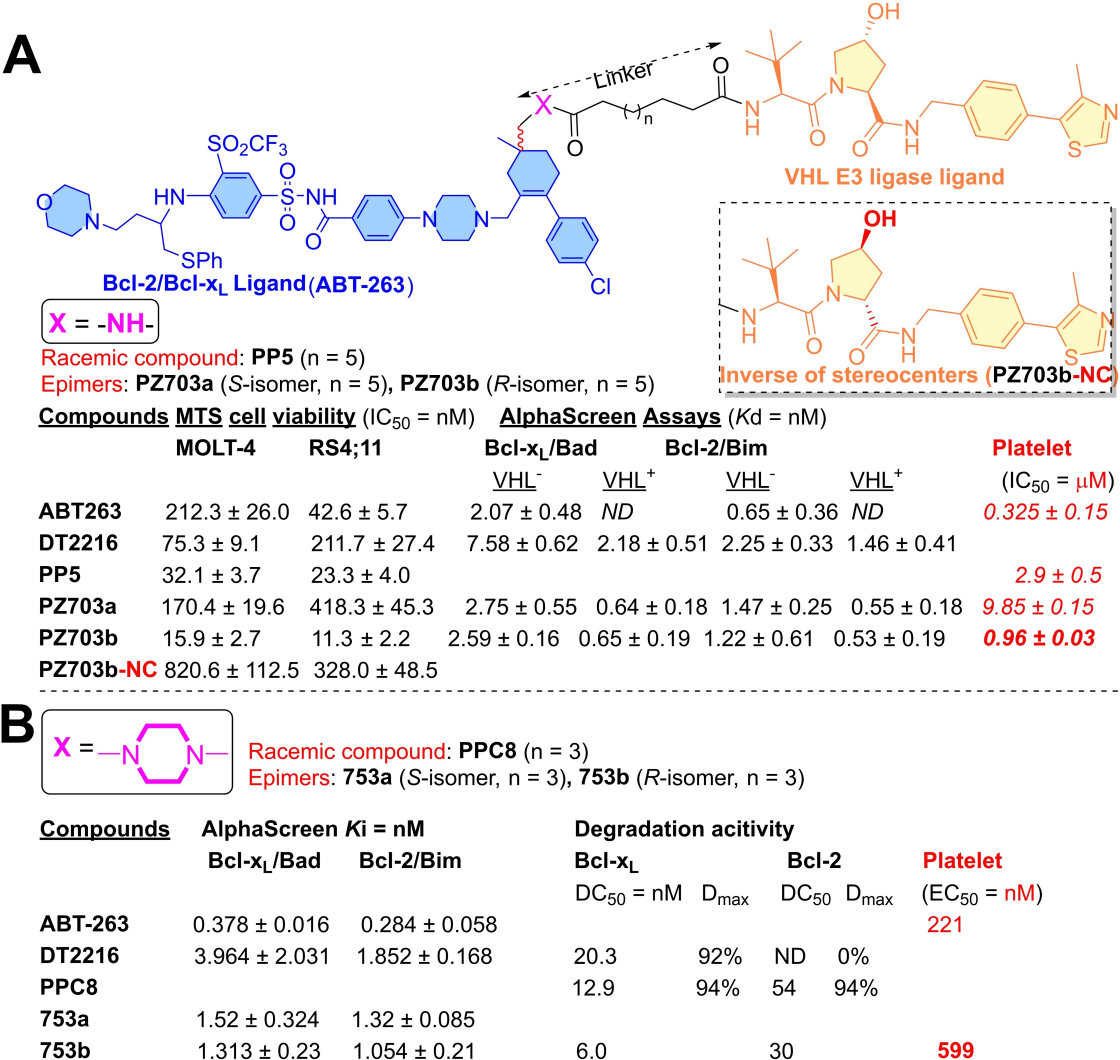
(A) PROTAC analogs derivatized ABT‐263 and VHL E3‐ligase ligand. VHL^−^=without VHL; VHL^+^=with VHL. (B) PROTAC analogs derivatized ABT‐263 and VHL E3‐ligase ligand.

To improve the anticancer activity spectrum of **DT2216**, the researchers attempted to enhance its ternary complex stability with Bcl‐2 protein. The co‐crystal structure of **ABT‐263** with Bcl‐2 (PDB code: 6QGH) was studied, where dimethyl groups of cyclohexene substructure of **ABT‐263** were found in the solvent‐exposed region. This information helped the authors to find another tethering point on **ABT‐263** to attach a VHL ligand (as shown in Figure 7A).^[37]^ Using dimethyl group of cyclohexene as tethering point, the linker length was exploited to synthesize eight PROTACs (**PP1**‐**8**). Based on cell viability assays on MOLT‐4 and RS4;11 cancer cell lines (as shown in Figure 7A), **PP5** was found the most potent cytotoxic among these PROTACs. The densitometric analysis of Bcl‐x_L_ expression in MOLT‐4 and RS4;11 cancer cell lines, found a two‐fold degradation potency of **PP5** over **DT2216** (DC_50_ values are shown in Table 2).^[37]^ As **PP5** was a racemic mixture, therefore was resolved into its epimers: *S*‐epimer (**PZ703a**) and *R*‐epimer (**PZ703b**). The cell viability assays on MOLT‐4 and RS4;11 cancer cell lines showed a significant potency of R‐epimer (**PZ703b**) over S‐epimer (**PZ703a**), and a two‐fold more potent than parent PROTAC (**PP5**). Such differences in activity among epimers, also exemplify a critical role of stereochemistry in shape recognition at the ligand‐protein interface in drug design. To understand the mechanism, competitive assays were performed on MOLT‐4 cells. MOLT‐4 cells were preincubated with **MG‐132** and VHL‐032 (a VHL ligand)^[38]^ and then treated with **PZ703b**. This resulted in a decrease in the Bcl‐x_L_ degradation, indicating that **PZ703b** utilizes an E3 ligase‐dependent proteasomal degradation of Bcl‐x_L_ protein. Additionally, a negative control (**PZ703b‐NC**) was developed with a synthetic strategy that was used for **DT‐2216‐NC**. As excepted, **PZ703b‐NC** showed the lowest cellular cytotoxicity than the reported PROTACs in their study (**PP1**‐**8**, both epimers of **PP5** and **DT2216**), as shown in Figure 7A. By cell‐free AlphaLISA assay, the *in‐vitro* ability of ternary complex formation of PROTACs (**DT2216**, **PZ703a**, and **PZ703b**) was evaluated. **PZ703b** showed a strong signal, while all formed *in‐vitro* ternary complex with Bcl‐x_L_ and Bcl‐2 but not with Mcl‐1 (an antiapoptotic member of Bcl‐2 protein family). By NanoBRET assay, **PP5**, **PZ703a**, and **PZ703b** were shown to form stable ternary complexes, while **PZ703b** showed a most pronounced effect in live cells. However, **DT2216** and **PZ703b‐NC** were unable to form ternary complexes in live cells. Surprising, the most potent epimer showed a higher platelet affinity (**PZ703b**, IC_50_=0.96 μM) than other epimer (**PZ703a**, IC_50_=9.85 μM) and its parent PROTAC (**PP5**, IC_50_=2.9 μM).^[37]^

**Table 2:** Bcl‐x_L_ degradation by PP5‐based PROTACs in MOLT‐4, RS4;11.

VHL‐based Bcl‐x_L_ PROTACs	Bcl‐x_L_ expression in cancer cell types^[a]^ (DC_50_=nM)	
	MOLT‐4	RS4;11
**DT2216**	68.6 ±15.1	58.3 ±6.3
**PP5**	31.6 ±7.2	22.6 ±6.6
**PZ703a**	127.4 ±27	75.2 ±9.5
**PZ703b**	14.3 ±5.1	11.6 ±3.2

[a] Densitometric analysis.

Optimization on the molecular frame of **PP5** led to the development of another class of Bcl‐x_L_‐PROTACs with much enhanced Bcl‐2 degradation activity.^[39]^ The dimethyl groups of cyclohexene structure of **ABT‐263** were extended into a piperazine ring that was tethered to a VHL ligand (as shown in Figure 7B). Exploration of the linker length led to the synthesis of seven PROTACs (**PPC5**‐**11**). Based on Bcl‐x_L_/Bcl‐2 degradation studies in HEK293T cells, **PPC8** showed maximum degradation (94 %) for both the proteins (D_max_ values are shown in Figure 7B). As **PPC8** was a racemic PROTAC, that was resolved into its epimer (*S*‐**753 a** and *R*‐**753 b**). Like the chemical biology of **PP5** (where *R* epimer **PZ703b** showed higher potency than *S*‐epimer), similar observations were also found with the epimers of **PPC8**. The *R*‐epimer (**753 b**) showed a higher Bcl‐2/Bcl‐x_L_ degradation activity than its parent PROTAC (**PPC8**). Interestingly, the Bcl‐2/Bcl‐x_L_ binary binding affinities for both epimers were found weaker than **ABT‐263**. The stability of the ternary complex of **753 a**, **753 b**, and **DT2216** was studied with the help of AlphLISA (*in‐vitro*) and NanonBRET (in live cells) assays. **753 b** shown a stronger *in‐vitro* ternary complex stability with Bcl‐x_L/_Bcl‐2 than **753 a** and **DT2216**. Similar results were also obtained from NanoBRET assays (except no ternary complex was detected for **DT2216** with Bcl‐2 protein). In the NanoBRET assay, *R*‐**753 b** showed a higher affinity in forming the ternary complexes with Bcl‐x_L_ and Bcl‐2 proteins than *S*‐**753 a**. Further experiments on VHL‐knock out models and preincubated cells with proteasome inhibitors (**MLN4924** and **MG132**), abrogated the Bcl‐x_L_ as well as Bcl‐2 degradation activity of *S*‐**753 b**, indicating the proteasomal mediated Bcl‐2/Bcl‐x_L_ degradation mechanism. Compared to the other dual Bcl‐x_L_/Bcl‐2 degrader (**Pz703b**) as shown in Figure7 A, **753 b** shows a relatively lower therapeutic index towards platelets.^[39]^

**Figure 8:**
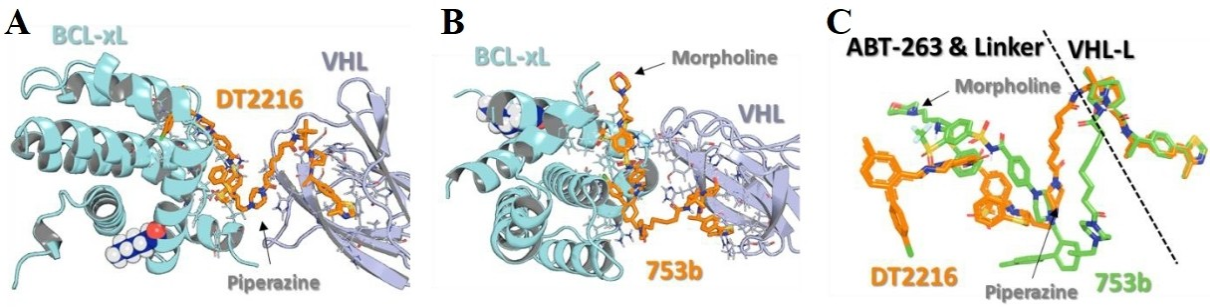
Ternary complexes of DT2216 (A), 753b (B), and superpose of **DT2216**/**753 b** (C). Reproduced with permission from Lv et al.^[39]^ Copyright 2021 Nature Publishing Group.

The ternary complex models of **753 b** and **DT2216** were studied (as shown in Figure 8). In comparison, more residues at the interface of Bcl‐x_L_: VHL proteins were interacting with the binding conformation of **753 b** (Figure 8B) than **DT2216** (Figure 8A). In corresponds to the piperazine ring of **DT2216**, the morpholine ring of the **753 b** was found significantly offset (as shown in Figure 8C), suggesting the adopted binding conformation of **DT2216** deviated from the original binding conformation of **ABT‐263**, possibly during its transformation to the PROTAC structure. However, **753 b** showed a similar binding mode of **ABT‐263** and explained why **753 b** is more potent PROTAC than **DT2216**.

**Figure 9:**
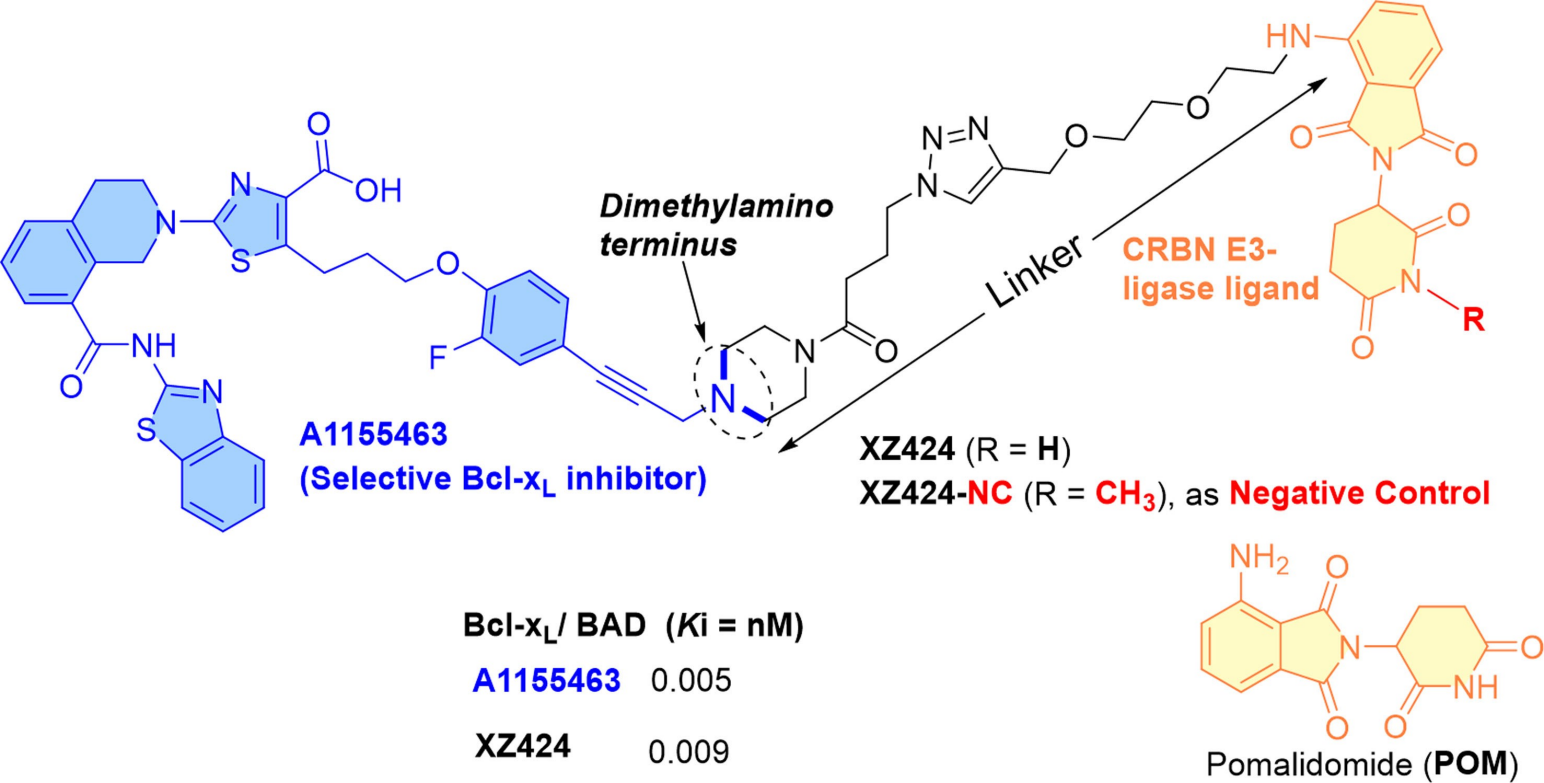
Chemical structure of **A1155463** based PROTAC with CRBN E3 ligase.

Studies reported cereblon (CRBN) as one of the E3 ligase other than VHL E3 ligase, which is commonly expressed in various cancer cell types but less in platelets.^[40]^ This information led researchers to develop CRBN E3 ligase‐based PROTACs to reduce the on‐target platelet toxicity of Bcl‐x_L_ inhibitors. The co‐crystal structure of Bcl‐x_L_ with **A1155463** was studied, where the dimethylamino terminus of **A1155463** was found in the solvent‐exposed region (highlighted in blue color in Figure 9). With the application of azide‐alkyne Huisgen cycloaddition^[41]^ (a common synthetic strategy that is used for macrocyclization or conjugating the warheads), the dimethylamino terminus of **A1155463** was tethered with a CRBN E3 ligase ligand (pomalidomide),^[40]^ which resulted in the synthesis of **XZ424**. By Alpha screen binding assay, a similar picomolar Bcl‐x_L_ inhibition range for **XZ424** (*K*i=9 pM) and **A1155463** (*K*i=5 pM) was observed. In MOLT‐4 cells,^[42]^ **XZ424** exhibited a dose‐dependent Bcl‐x_L_ degradation (DC_50_=50 nM under 16 h treatment) but no change was observed in Bcl‐x_L_ protein level in platelets (up to 1.0 μM for 16 h). The dose‐response studies showed a Bcl‐x_L_ degradation of **XZ424** was initiated after 2 h and reached >85 % degradation after 16 h (at 100 nM) in MOLT‐4 cells. Later, MOLT‐4 cells were preincubated with **MG132** and an excess of the pomalidomide to evaluate the CRBN mediated proteasomal mechanism of **XZ424**. As expected, the activity of **XZ424** was severely reduced in these assays, which supported the utilization of the CRBN mediated ubiquitination for its Bcl‐x_L_ degradation activity. No activity was observed for its negative control (**XZ424‐NC**, which is a methylated version of **XZ424** as shown in Figure 9) in MOLT‐4 cells, which confirms the CRBN mediated Bcl‐x_L_ degradation of **XZ424**. Interestingly, **A‐1155463** (EC_50_=6.2±4.3 nM at 72 h) and **XZ424** (EC_50_=51±23 nM at 72 h) showed a nanomolar range MOLT‐4 cytotoxicity while a significant platelet toxicity difference between **A‐1155463** (EC_50_=7.1±2.6 nM at 24 h) and **XZ424** (EC_50_=1136±27 nM at 72 h) was observed. In comparison, **XZ424** lost some Bcl‐x_L_ affinity during its transformation from **A‐1155463** but maintained a nanomolar range Bcl‐x_L_ inhibition with significantly improved therapeutic index to platelets (a twenty‐two times selectivity of **XZ424** versus no selectivity of **A‐1155463**). The observed differences in anticancer and platelet activities reflect a key role of the molecular obesity of **XZ424** in decreasing its platelet cell permeability. Additional experiments (western blotting and flow cytometry) indicated that **XZ424** induces caspase‐dependent apoptosis in MOLT‐4 cells.^[40]^

**Figure 10:**
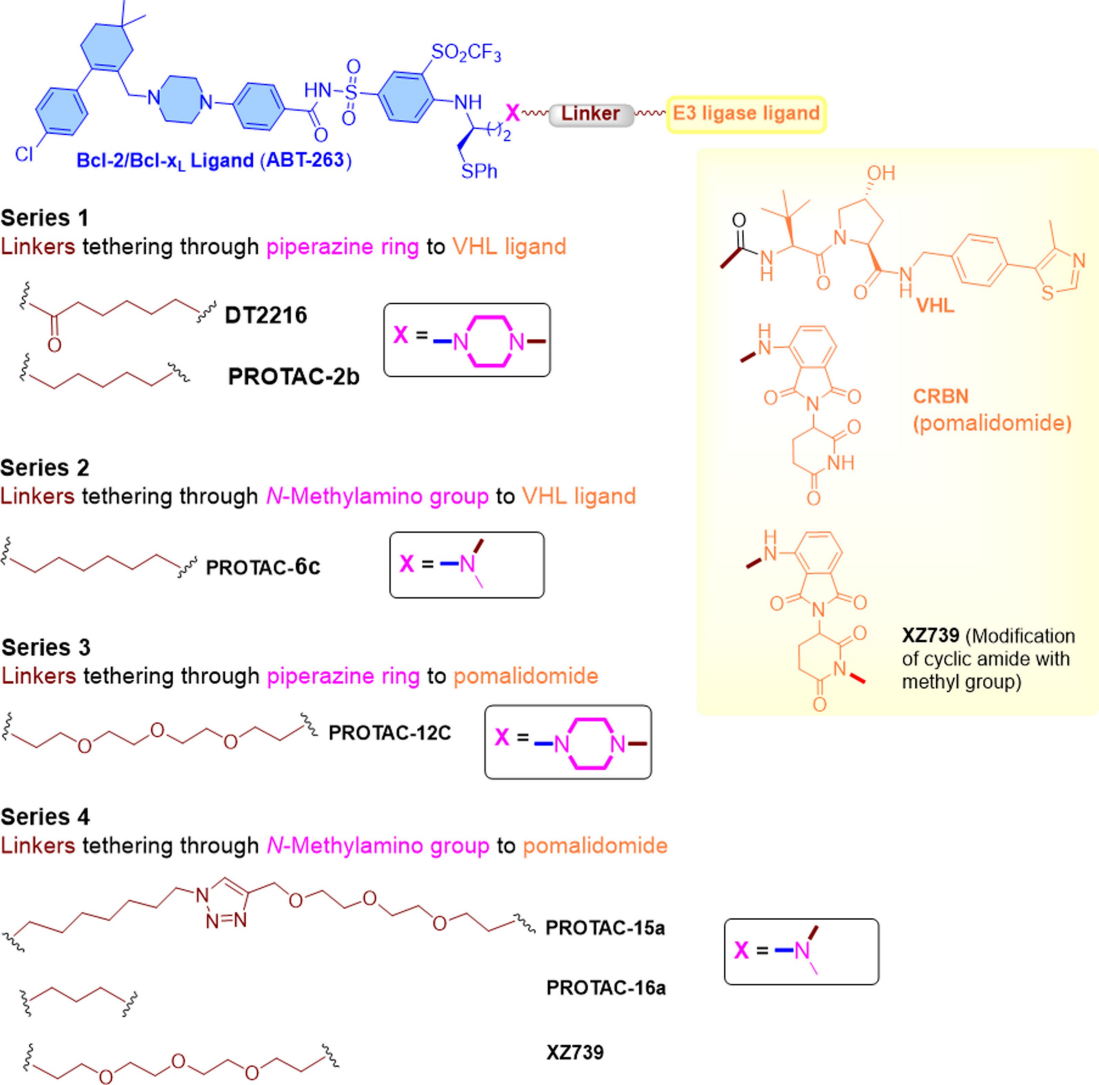
Bcl‐x_L_ degraders: Investigation of linker chemical space and tethering points to the warheads.

To expand the scope of structure‐activity relationship (SAR) studies, the authors explored the different nature of linkers (aliphatic, polyethers, triazole ring‐embedded), tethering points on **ABT‐263** and E3 ligase ligands (VHL/CRBN).^[43]^ The tethering point on **ABT‐263** for the linkers was either piperazine ring (**series 1** & **3**) or *N*‐methylamino functionality (**series 2** & **4**), as shown in Figure 10. While series 1–4 utilized VHL (**series 1** & **2**) or CRBN (**series 3** & **4**) as E3 ligase ligands, as shown in Figure 10. The cell viability and estimated degradation rates of the potent PROTACs from each **series 1**–**4** are compiled in Table 3. The polyether linker containing **XZ739** ((DC_50_=2.5 nM)) from **series 4**, which has an *N*‐methylamino functionality as a tethering point on **ABT‐263** and CRBN E3‐ligase ligand, was found 21‐times more potent Bcl‐x_L_ degrader than **DT2216** (DC_50_=53 nM).^[43]^ In MOLT‐4 cells, degradation started at 2 h, and more than 96 % was observed after 8 h at 100 nM of **XZ739**. The washout experiments found Bcl‐x_L_ degradation, long‐lasting and reversible. The effect of **XZ739** on the cellular level of proteins in MOLT‐4 cells was determined by western blotting, which suggested a dose‐dependent Bcl‐x_L_ degradation. Preincubation with a proteasome inhibitor (**MG‐132**) and, excess of CRBN ligand (pomalidomide) abrogated the Bcl‐x_L_ degradation activity of **XZ739** in MOLT‐4 cells, supporting the CRBN E3 ligase mediated proteasomal degradation. To confirm the Bcl‐x_L_ degradation mechanism of **XZ739**, **XZ739‐NC** was developed as a negative control. Chemically, **XZ739‐NC** has a structure like **XZ739**, with a methyl group substituted on the cyclic amide (‐NH‐) of the pomalidomide to prevent its binding to E3 ligase (as shown in Figure 10). As anticipated, no Bcl‐x_L_ degradation was observed with **XZ739‐NC**, which confirmed the CRBN‐mediated Bcl‐x_L_ degradation mechanism of **XZ739**. In comparison to **ABT‐263**, **XZ739** showed a preferential cell‐selectivity (MOLT‐4 than RS4;11 or H146 cell lines) (as shown in Table 3), and with a 120‐folds less cytotoxic to platelets.^[43]^

**Table 3:** Most potent Bcl‐x_L_ PROTACs degradation with diverse linker chemotypes.^[43]^

Bcl‐x_L_ PROTACs	IC_50_ (nM)^[a]^ 48 h treatment				DC_50_^[b]^	IC_50_ ratio^[c]^
	MOLT‐4	RS4; 11	H146	Platelets		
**ABT‐263**	230*	49	43.8	242	ND	1.1
**DT2216**	77.1	213	278	>10,000	53	>130
**PROTAC‐2b**	82	76	203	>10,000	93	>122
**PROTAC‐6c**	81.3	189	265	>10,000	71	>123
**PROTAC‐12c**	17.3	38.5	24.6	1560	4.5	90
**PROTAC‐15a**	29.2	62.2	61.7	6250	6.3	214
**PROTAC‐16a**	32.5	129	70.2	3296	10.6	101
**XZ739**	10.1	41.8	25.3	1217	2.5	120

[a] IC_50_ values are the means of at least three independent experiments. [b] In MOLT‐4 cells, 16 h treatment. [c] IC_50_ ratio between human platelets and MOLT‐4 cells. * typographical error: different values for **ABT‐263** (IC_50_=230 nM in Table 1; IC_50_=227 nM in Table 5) mentioned in original paper.^[43]^

### 3.3 Bcl‐x_L_ targeted PROTACs in senescence cells

**Figure 11:**
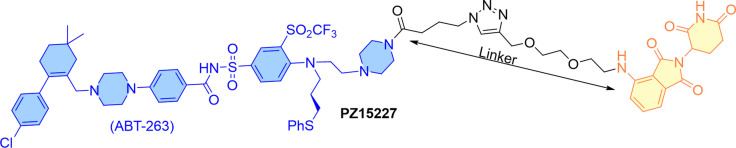
**PZ15227**: ABT‐263 warhead (Bcl‐XL) that recruits CRBN E3 ligase.

As the survivability of senescent cells is commonly related to the overexpression of Bcl‐x_L_ protein, therefore the application of Bcl‐x_L_ protein inhibitors as senolytic agents certainly has clinical significance. Zheng and Zhou's co‐worker explored this opportunity of using Bcl‐x_L_ PROTACs for targeting the senescent cells (SC).^[44]^ They developed **PZ15227**, a CRBN‐recruiting degrader based on the **ABT‐263** structure (as shown in Figure 11). Using Alphascreen binding assay, **PZ15227** (*K*i: Bcl‐x_L_/Bad=1.90±0.15 nM, Bcl‐2/Bad=3.52±0.26 nM, Bcl‐w/Bad=>1000 nM) exhibited Bcl‐x_L/_Bcl‐2 binding affinities similar to **ABT‐263** (*K*i: Bcl‐x_L_/Bad=1.53±0.07 nM, Bcl‐2/Bad=1.03±0.11 nM, Bcl‐w/Bad=8.5 nM). As the survival of senescent cells depend on the expression of Bcl‐x_L_ but not on Bcl‐2/Bcl‐w, therefore the cellular level of these proteins in non‐senescent cells (WI38 human fibroblast cells) were quantified by using western blotting in a dose‐dependent manner. In these experiments, **PZ15227** showed a rapid long‐lasting Bcl‐x_L_ degradation (DC_50_=0.046 μM; D_max_=96.2 %) and >65‐times potency than its Bcl‐2 degradation (DC_50_=>3 μM; D_max_=36.1 %) and Bcl‐w (DC_50_=>3 μM; D_max_=16 %).^[44]^ A similar degradation profile of **PZ15227** was also observed for WI138 senescent cells ( WI138 SCs). To evaluate the platelet toxicity, **PZ15227** was compared with **ABT‐263** using MTS assays at 24, 48, and 72 h intervals on mouse platelet cells. The resulted EC_50_ values at 24 h (**ABT‐263**=1.02 μM; **PZ15227**=>10 μM), 48 h (**ABT‐263**=0.39 μM; **PZ15227**=>10 μM) and 72 h (**ABT‐263**=0.14 μM; **PZ15227**=3.32 μM), certainly showed a substantial improved platelet survivability from **Pz15227** than **ABT‐263**. In comparison to **ABT‐263**, **PZ15227** exhibited a slight improvement in Bcl‐x_L_ potency for WI138 SCs. To widen its senolytic spectrum, cell viability assays were performed on various types of senescent cell lines: WI38 replicative senescent cells (EC_50_: **ABT‐263**=1.23 μM, **PZ15227=**0.13 μM), WI38 Ras oncogene‐induced senescent cells (EC_50_: **ABT‐263**=0.49 μM, **PZ15227**=0.61 μM), IMR90 ionizing radiated senescent cells (EC_50_: **ABT‐263**=0.34 μM, **PZ15227**=0.30 μM), REC ionizing radiated senescent cells (EC_50_: **ABT‐263**=0.52 μM, **PZ15227**=0.29 μM), PAC ionizing radiated senescent cells (EC_50_: **ABT‐263**=0.28 μM, **PZ15227**=0.074 μM). These EC_50_ values clearly showed **PZ15227** as a broad‐spectrum senolytic agent. Results from other experiments further verify the **PZ15227** as a selective Bcl‐x_L_ degrader but not a Bcl‐2 degrader, such as (a) no reasonable change in the cellular levels of Bcl‐2 or Bcl‐w proteins was observed, (b) no Bcl‐2 poly‐ubiquitination was recorded, (c) a moderate‐to‐low change in the glutamate synthetase expression.^[45]^ Additional observations indicated the **PZ15227** follow a CRBN‐dependent proteasomal degradation mechanism, such as (a) no Bcl‐x_L_ degradation was recorded with preincubated cells with **ABT‐263** or pomalidomide or **MG132**, (b) no change was found in the cellular level of Bcl‐x_L_ with CRBN knock out cells, (c) methylation on pomalidomide substructure of **PZ15227** abrogated its Bcl‐x_L_ degradation activity. Later, pharmacokinetic studies of **PZ15227** were performed in naturally aged mice models. Compared to the **ABT‐263**, **PZ15227** showed reasonable metabolic stability (plasma as well as microsomal) and bioavailability (intraperitoneal and intravenous than the oral route), with less aqueous solubility. By intraperitoneal route, **PZ15227** showed moderate thrombocytopenia compared to an equivalent effective dose of **ABT‐263** (41 μmol/kg) in the mice models. Whereas the first dose of **ABT‐263** (41 μmol/kg or 40 mg/kg) induced severe thrombocytopenia, while less intensive dosing of **PZ15227** (41 μmol/kg, per 3 days) exhibited a long‐lasting PROTAC activity than **ABT‐263**. Also, **PZ15227** was found to decrease significantly the splenic expression of key senescent cell biomarkers.

### 3.4 Bcl‐x_L_ targeted SNIPERS in cancer

**Figure 12:**
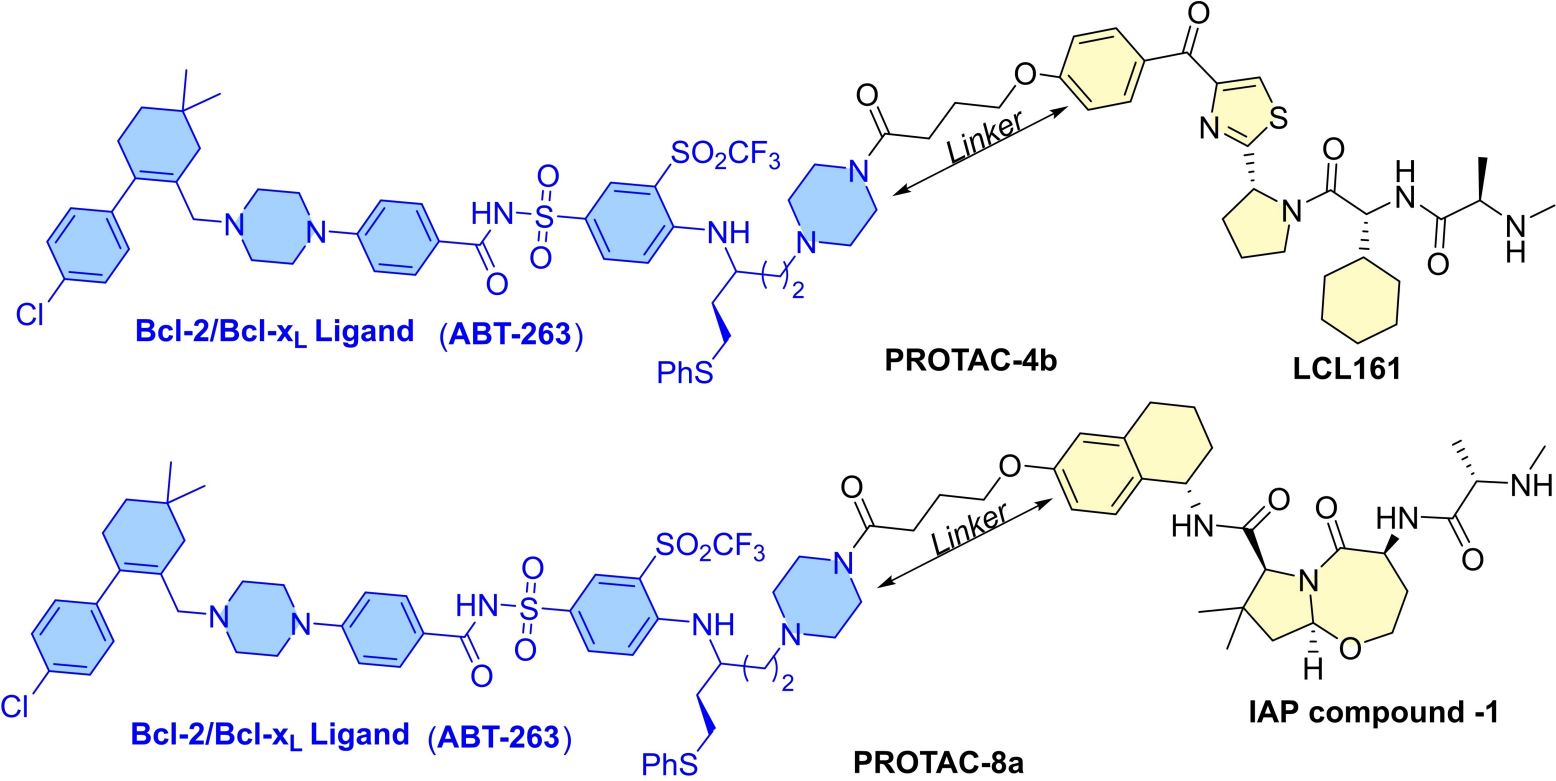
Chemical structure of PROTAC‐4b and PROTAC‐8a that recruits IAPs (LCL161 and IAP compound 1) for Bcl‐X_L_ degradation.

Two issues are commonly encountered in PROTAC strategies (a) mutation of E3 ligases and (b) a lower expression of E3 ligases in the target cells. Therefore, other event‐driven pharmacology‐based strategies were also investigated. In this aspect, Zheng and Zhou's co‐worker investigated IAP (inhibitor of apoptosis protein) based Bcl‐x_L_ degrader. In humans, eight IAP proteins are present, which have distinctive baculovirus IAP repeat (BIR) domain and a zinc‐binding domain in their three‐dimensional structure.^[4]^ Also, IAPs contain a Ub‐associated domain for binding to poly‐Ub chains and a RING (**r**eally **i**nteresting **n**ew **g**ene) domain that has E3 ligase activity.^[4]^ Studies showed a strong correlation of their overexpression or loss of endogenous antagonists of IAP proteins with cellular malignancy, survival, and poor prognosis. The PROTACs developed on IAP, are commonly called SNIPERs (**S**pecific and **n**on‐genetic **I**AP‐dependent **p**rotein **e**rasers) and are successfully developed for BCR‐ABL, bromodomain proteins, nuclear receptors (estrogen), and other proteins of interest. Similar to the **DT‐2216** synthetic strategy, authors developed **PROTAC‐4 b** (as shown in Figure 12) by replacing the morpholine ring of **ABT‐263** with a piperazine ring and used it as an attachment point with an XIAP antagonist (**LCL161**). XIAP (X‐linked inhibitor of apoptosis protein) is a protein called an inhibitor of apoptosis protein 3 (IAP3) and baculoviral IAP repeat‐containing protein 4 (BIRC4). XIAP binds to caspases (3, 7, and 9) and prevents cellular apoptosis. Similar to the other IAPs, it has a RING domain with E3 ubiquitin ligase activity, which activates the proteasomal ubiquitination of its own, caspase‐3, caspase‐7, and therefore, considered as the most potent human IAP protein. Some studies showed that the targeting of XIAP improves efficiency and can produce a large change in SNIPER‐induced proteasomal degradation.^[46]^ As **IAP compound 1** has a more potent XIAP binding affinity than **LCL161**, therefore authors developed **IAP compound 1** based **PROTAC‐8 a** (as shown in Figure 12). Reasonable cytotoxicity of **PROTAC‐1** against MyLa 1929 (T‐cell lymphoma cells, EC_50_=62 nM) compared to **ABT‐263** (EC_50_=50 nM) was recorded. In comparison to other PROTACs based on **ABT‐263** and CRBN E3‐ligase ligand (such as **XZ739** and **XZ424**), **PROTAC‐8 a** showed a high potency towards Myla 1929 and least potency towards MOLT‐4 cells. Preincubated cellular experiments with proteasomal inhibitor (**MG‐132**), **IAP compound‐1**, and **ABT‐263** confirmed a **PROTAC‐8 a** induced proteasomal degradation of Bcl‐x_L_ protein. As **IAP compound 1** has a high affinity for *m*e*l*anoma‐*IAP* (ML‐IAP, a IAP protein highly expressed in melanoma cells^[47]^) therefore, wild‐type and ML‐IAP knockout SK‐MEL‐28 cells were treated with **PROTAC‐8 a**. The purpose of this experiment was to evaluate the degradation mechanism of **PROTAC‐8 a**, which could be mediated through XIAP or ML‐IAP. However, if it would be ML‐IAP‐mediated, then a higher Bcl‐x_L_ degradation in wild type SK‐MEL‐28 cells (enriched with ML‐IAP protein) would be observed than knockout SK‐MEL‐28 cells.^[48]^ But, no distinctive difference in Bcl‐x_L_ degradation levels in both SK‐MEL‐28 cell variants was observed, which suggested **PROTAC‐8 a** utilize other IAP proteins than ML‐IAP. Even, no change in cellular levels of Bcl‐2 and Mcl‐1 protein was observed, indicating a higher Bcl‐x_L_ protein degradation specificity of **PROTAC‐8 a**. The cell viability assays on MyLa1929 cell line, showed **PROTAC‐8 a** with a nearly 1000‐folds cancer cell‐selectivity (IC_50_=62 nM, and IC_50_=8500 nM in platelets) while **ABT‐263** showed a moderate cell selectivity (IC_50_=50 nM; IC_50_=189 nm in platelets). The SAR studies indicated that the specificity of IAP ligands is crucial for its protein degradation activity, as **LCL161** derived PROTACs showed a moderate‐to‐low MyLa1929 activity compared to the **IAP compound 1** derived PROTACs. However, **LCL161** derived PROTACs with more than 8‐carbon linkers (polyether or triazole‐polyether) showed moderate submicromolar activity, while PROTACs with less than 8‐carbon length showed a decreasing cytotoxicity trend against MyLa 1929 cells. Interestingly, all PROTACs derived **IAP compound‐1** showed consistent cytotoxicity IC_50_ values less than 274 nM. The observed differences in the activities of these two different types of PROTAC certainly illustrate a high dependence on the specificity of IAP ligand, and therefore, a comprehensive profiling of IAP ligands must be done to understand their roles and expression at cellular level.

### 3.5 Prodrug strategies based on Bcl‐x_L_ inhibitors to reduce on‐target platelet toxicity

Besides protein degradation approaches mentioned in this manuscript (such as PROTAC and SNIPER), researchers also implemented prodrug‐based strategies to reduce the on‐target platelet toxicity of Bcl‐x_L_ inhibitors. The concept of prodrugs is used to minimize the drug exposure to the platelets and gets activated chemically or enzymatically into an active form when it reaches the targeted cells.

#### 3.5.1 Phosphate prodrugs based Bcl‐X_L_ inhibitors

**Figure 13:**
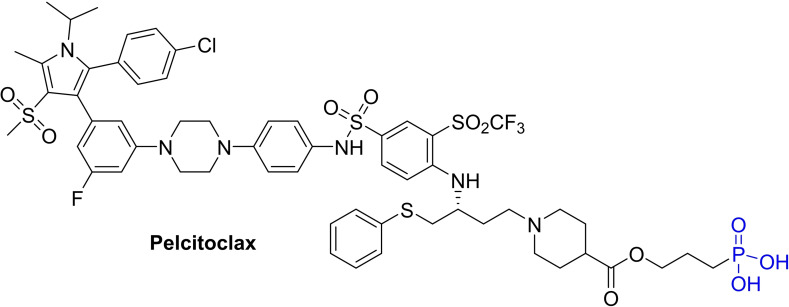
Chemical structure of Phosphate prodrug of Bcl‐xL inhibitors (**APG‐1252**).

A phosphate prodrug (**APG‐1252** (**BM‐1252**)) was developed on a dual Bcl‐x_L_/Bcl‐2 inhibitor, as shown in Figure 13.^[49]^ In comparison, **APG‐1252** showed lower cell‐permeability for the platelets than cancer cells. After reaching the target cells, **APG‐1252** gets converted into **APG‐1252‐M1** (**BM‐1252‐M1**) (an active form).^[49]^ Although **APG‐1252** and **APG‐1252‐M1** showed higher affinity for both proteins (Bcl‐2 and Bcl‐x_L_ in the range of *K*i<1 nM), **APG‐1252‐M1** showed ten‐fold cytotoxicity than **APG‐1252** in SCLC cells.^[49]^ Additionally, the **APG‐1252‐M1** demonstrated a Bax/Bak‐dependent apoptosis mechanism in MEF/MCL1^−/−^ cell line. Nevertheless, both forms (prodrug and active form) were able to achieve a complete tumor regression in animal cancer models, but **APG‐1252‐M1** showed an over 30‐folds platelet killing activity compared to its phosphate prodrug form (**APG‐1252**),^[49]^ which certainly relates a futuristic therapeutic application of such strategy to reduce on‐target toxicities of other therapeutic agents. Studies performed by Qiu and co‐workers at Sun Yat‐Sen University Cancer Center (Guangzhou, China) on gastric cell lines (six cell lines: AGS and N87, BGC‐823, SGC‐7901, MKN45, NUGC‐3) showed **APG‐1252‐M1** induced a time and dose‐dependent caspase‐3 activation in AGS and N87 cell lines.^[50]^ Their further experiments on xenograft models where they subcutaneously transplanted N87 cells into BALB/c athymic nude mice (male, 4 to 6weeks), resulted in tumor suppression. With colorectal cancer cell lines, **APG‐1252‐M1** showed a nanomolar range as a single agent, while synergistic activity with **ortrametinib**. Additionally, in combination with **gemcitabine**, **APG‐1252** showed an activation of the caspase cascade, downregulation of the JAK‐2/STAT3/Mcl‐1 axis, and epithelial‐mesenchymal transition, that produces a synergistic effect in advanced nasopharyngeal carcinoma cells (CNE2, HNE1, and TW03).^[51]^ In AML cell testing, **APG‐1252‐M1** (APG‐1252‐12 A) exhibited Bcl‐2/Bcl‐x_L_ mitochondria‐dependent apoptosis.^[52]^ The current ongoing trials^[53]^ on **APG‐1252** (commercial name: **Pelcitoclax**) are summarized in Table 4.

**Table 4:** Status of clinical phase and related studies.

Trial number	Clinical condition or implications	Clinical study phase
NCT03387332	SCLC and other solid tumors	Phase‐I
NCT04210037	SCLC	Phase‐I/II
NCT04001777	EGFR^+^ NSCLC	Phase‐I
NCT04893759	neuroendocrine tumors	Phase‐I
NCT03080311	SCLC, solid tumor	Phase‐I
NCT04354727	myelofibrosis	Phase‐I/II

SCLC: small cell lung cancer; NSCLC: non‐small cell lung cancer.

#### 3.5.2 Antibody‐Bcl‐X_L_ inhibitor conjugate

This strategy is commonly called **A**ntibody‐**D**rug **C**onjugate (ADCs), which efficiently delivers the active drug onto the site. This way, the possible risk of drug exposure to the normal tissues (in this case, platelet cells) gets reduced and improves the therapeutic index of the drug. The phase‐I clinical trial of the Bcl‐x_L_‐targeting antibody‐drug conjugate (ADC), **ABBV‐155** (**mirzotamab clezutoclax**) was commenced on July 13, 2018, which is expected to get completed on September 29, 2022. In this clinical investigation (clinical trial: NCT03595059), **ABBV‐155** will be investigated as a single‐acting agent and, in combination with **paclitaxel** in adult patients with relapsed and refractory solid tumors.^[54]^

#### 3.5.3 Bcl‐x_L_ dendrimer conjugate

**Figure 14:**
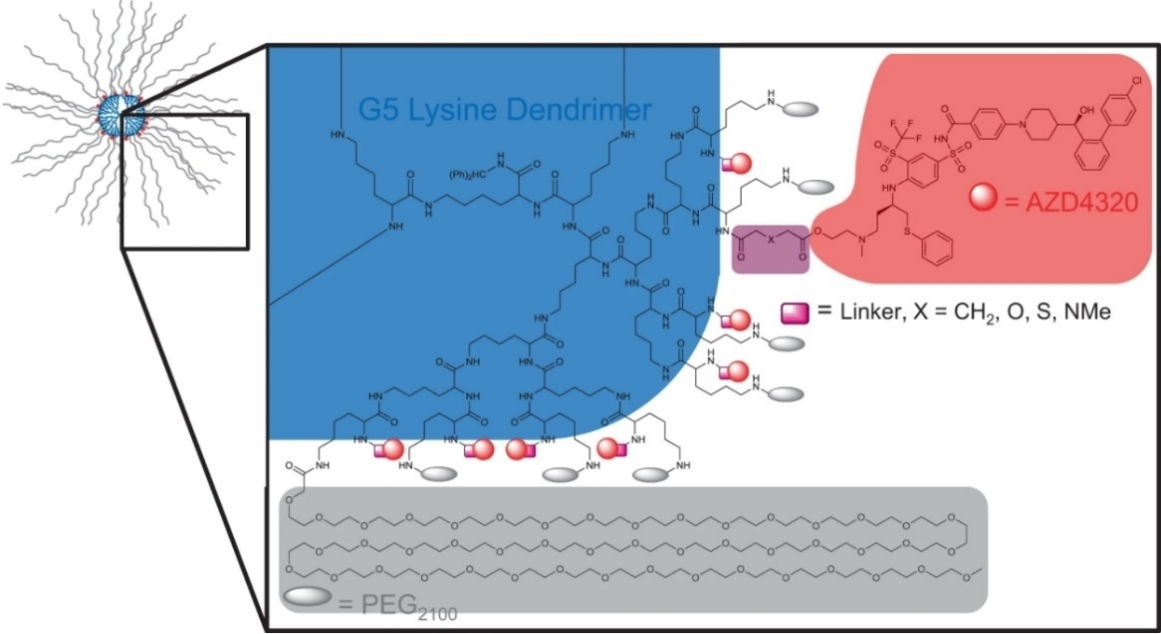
Representation of **AZD4320‐**dendrimer conjugate: Each dendrimer presenting 32 PEG2100 terminals (in grey color), 32 **AZD4320** (in red color) linker regions (marked in pink color), where if X=CH_2_ for **SPL‐8931**, X=S for **SPL‐8932** and X=O for **SPL‐8933**.^[55]^ Reproduced with permission from Patterson et al^[55]^ Copyright 2021 Nature.

A collaboration of research teams of AstraZeneca from Bollington, United Kingdom, and Boston (USA) developed **AZD0466**. The **AZD0466** is a dendrimer conjugate, where a dual inhibitor (**AZD4320**) of Bcl‐x_L_/Bcl‐2 proteins is conjugated with PEGylated poly‐lysine units, as shown in Figure 14.^[55]^ The initial use of **AZD4320** was to achieve a rapid intravenous Bcl‐2/Bcl‐x_L_ inhibition in a wide range of cancers with manageable on‐target platelet toxicity. However, cardiotoxicity, poor aqueous solubility (<1μg/ml), and high plasma protein binding of **AZD4320** during preclinical development discouraged its further development. Later, the low therapeutic index of **AZD4320** was addressed by developing its dendrimer‐based drug delivery, which improved its delivery to the target cells. Based on the linker‐atom type, the following dendrimers (**SPL‐8931**, X=S for **SPL‐8932** and X=O for **SPL‐8933**) were developed, as shown in Figure 14. The objective of developing such specific linker‐atom type dendrimers was to broaden the pharmacokinetic studies compared to the therapeutic efficacy and tolerability of **AZD4320**. The release rates for **AZD4320** from all the three dendrimers (**SPL‐8931**, **SPL‐8932**, and **SPL‐8933**) showed first‐order kinetics with T_1/2_ 201, 4.4, and 1.7 h, respectively. In the mouse xenograft model, all dendrimers had similar pharmacokinetics where total plasma concentration was cleared by reticuloendothelial system uptake. Using mathematical modeling,^[55]^ the optimal release rate of **AZD4320** was estimated for the maximal therapeutic index to anticancer efficacy and cardiovascular tolerance. In a dog telemetry study, **AZD0466** showed a reversible dose‐dependent decrease in platelet number, where recovery was achieved during its subsequent dosing. Although this study doesn't directly relate the Bcl‐x_L_ associated platelet toxicity but elucidates a possible alternative strategy for Bcl‐x_L_ targeting with manageable platelet toxicity. As dendrimeric‐form (**AZD0466**) of **AZD4320**, showed an improvement in therapeutic index and dose‐dependent cardiotoxicity, which encouraged the researchers to evaluate the **AZD0466** for its phase I clinical trial (clinical trial: NCT04214093).^[56]^ The preliminary clinical results of **AZD4320** showed potent cytotoxicity in mantle cell lymphoma cells (IC_50_=1.6–78 nM).^[57]^ In an independent study, a synergism of **AZD4320** with **acalabrutinib** showed a suppression of the cell proliferation in ibrutinib/venetoclax‐sensitive and ‐resistant cell lines (combination index=0.17–0.93).^[57]^

#### 3.5.4 Galacto‐conjugation of Bcl‐x_L_ inhibitors

**Figure 15:**
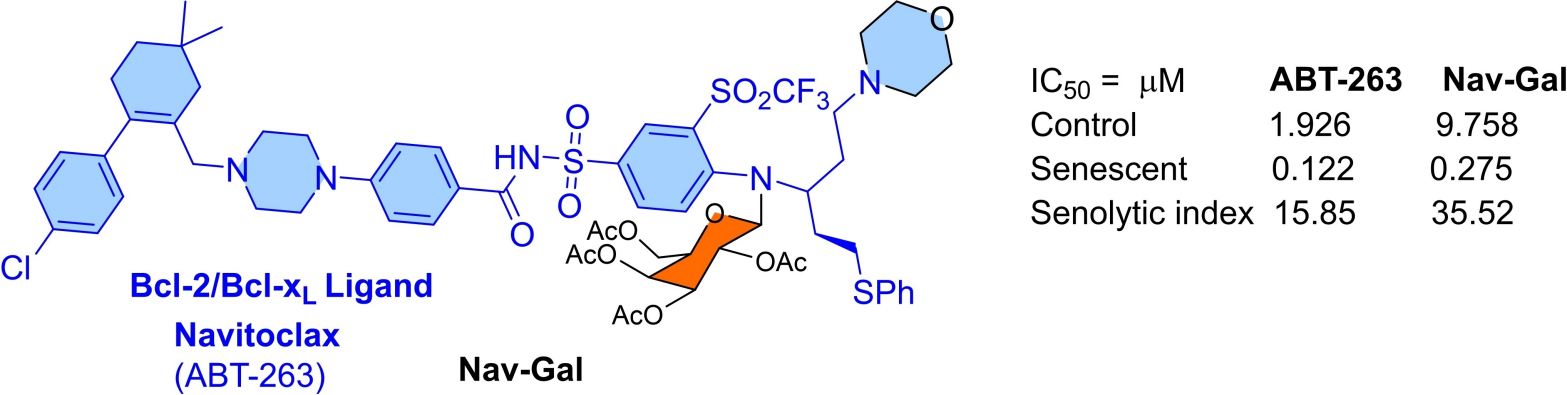
**ABT‐263**‐derived Nav‐Gal structure.

Authors exploited the higher expression of a senescence‐associated lysosomal *β*‐galactosidase (SA‐β‐gal) enzyme in senescent cells to improve the therapeutic index of Bcl‐x_L_ inhibitors.^[58]^ Collaboratory work of Mánez (Universität Politècnica de València, Valencia, Spain) and colleagues demonstrated a prodrug strategy for senolytic agent with reduced platelet toxicity.^[58]^ They developed a galacto‐conjugate prodrug (**Nav‐Gal**) based on the **ABT‐263** structure, as shown in Figure 15. The **Nav‐Gal** is a potent senolytic prodrug activated by lysosomal SA‐β‐gal activity in a wide range of cell types. Therapy‐induced senescence cell viability assays were used to evaluate **Nav‐Gal** effectiveness. In these assays, the lung cancer cell line (A‐549) was initially pretreated with **cisplatin** for ten days. The cisplatin‐pretreatment increased the expression of SA‐β‐gal and senescence biomarkers. Later, **cisplatin‐**pretreated cells were treated with an increasing dose of **ABT‐263** and **Nav‐Gal**. After 72 h of treatment, the IC_50_ values for **ABT‐263** (0.122 μM) and **Nav‐Gal** (0.275 μM) were calculated. However, senolytic index for **ABT‐263** and **Nav‐Gal** were found 16‐folds and 36‐folds, respectively. Further studies utilized the GLB1 gene (a gene responsible for the expression of SA‐β‐gal enzyme in the cells) knock‐down cancer cell models (A549 and SK‐Mel‐103) to evaluate the senolytic effect of **Nav‐Gal** on lysosomal SA‐β‐gal activity of senescent cells. A decrease in senolytic activity for **Nav‐Gal** and no activity change for **ABT‐263** indicated a SA‐β‐gal enzyme‐dependent senolytic activity of **Nav‐Gal**. In conclusion, **Nav‐Gal** was found as a selective‐apoptosis inducer in senescent cells than non‐senescent cells, with a higher senolytic index than its parent compound (**ABT‐263**) (comparative values shown in Figure 15). In combination with **cisplatin**, **Nav‐Gal** showed an additive antitumor effect in lung cancer cells (A549 cell line). Furthermore, to validate the *in‐vivo* efficacy of **Nav‐Gal** in combination with senescence‐inducing chemotherapy, authors developed a mice model (where A549 cells were transplanted subcutaneously into severe combined immunodeficient mice). Histological data showed a reduced level of p21 and Ki67, suggesting apoptosis of senescent cells facilitate the antitumor effect. In further studies, an orthotopic model of NSCLC was used, where wild type‐C57BL/6 J mice were transplanted with syngenic luciferase‐expressing KP lung adenocarcinoma cell line (L1475luc). The histological analysis demonstrated a high effectivity of combination therapy of cisplatin with **Nav‐Gal** in preventing *in‐vivo* tumor growth. Comparative studies of **Nav‐Gal** with **ABT‐263** showed reduced on‐target platelet toxicity in *ex‐vivo* (human and murine blood samples) and *in‐vivo* (wild type‐C57BL/6 J) models. The mode of the mechanism of **Nav‐Gal** exhibited its passive update into the non‐senescent and senescent cells. As senescent cells have higher expression of β‐galactosidase than non‐senescent cells, therefore a high percentage of hydrolysis of the glycosidic bond of **Nav‐Gal** occurred in senescent cells that release a higher amount of **ABT‐263** and inhibits the Bcl‐x_L_ protein.

## 4 Conclusion and Future Perspective

**Figure 16:**
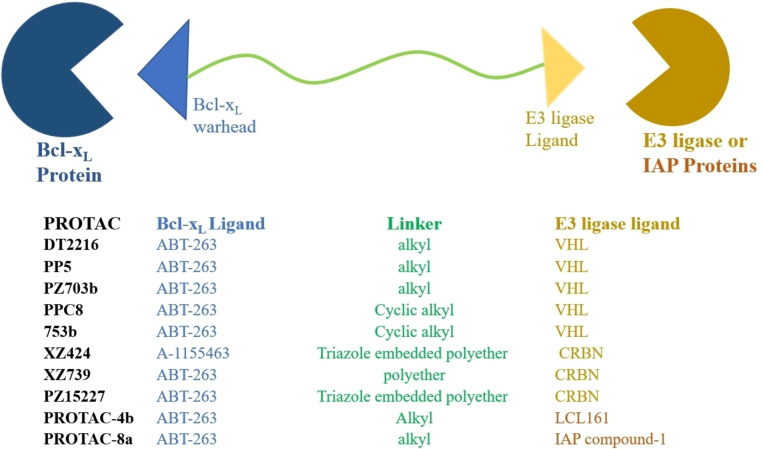
Summarized structures of Bcl‐x_L_ PROTACs and SNIPERs.

Apoptosis is a tightly regulated cellular process, and its evasion is linked to the survival of cancer and senescent cells. The evasion of apoptosis is often correlated with an abnormal expression of intracellular antiapoptotic proteins. Bcl‐x_L_ is an antiapoptotic protein that prevents apoptosis and, is considered an attractive target for cancer and senolytic therapies. On the other hand, a higher expression of Bcl‐x_L_ in solid tumors and, to some leukemia and lymphomas is reported and, further linked to the acquired resistance of some of the conventional anticancer therapeutics (especially cyclophosphamide, doxorubicin, and taxols).^[59]^ Although various Bcl‐x_L_ inhibitors were developed in recent years, most of them were suffered from on‐target and dose‐dependent platelet toxicities as an expression of Bcl‐x_L_ is essential for platelet survival. Even, **ABT‐263** failed in clinical trials due to its on‐target platelet toxicity. Initially, synergistic combinations with Bcl‐x_L_ inhibitors were attempted, such as **ABT‐263** with **JQ‐1** (bromodomain inhibitor) and **ABT‐263** with **docetaxel**. These combinations allowed to decrease the effective dose of Bcl‐x_L_ inhibitor and thereby reduce their dose‐dependent toxicities. Because of the limited number of clinical agents that can be used with Bcl‐x_L_ inhibitors, other strategies were also investigated (mainly included, (a) Bcl‐x_L_ targeted PROTACs, (b) Bcl‐x_L_‐targeted SNIPERS, (c) Prodrugs‐based Bcl‐x_L_ inhibitors). As most Bcl‐x_L_ inhibitor designs were based on occupancy‐driven pharmacology, therefore frequent‐dosing with picomolar‐to‐nanomolar range inhibitors is often required for continuous therapy. Recent interests in developing the strategies based on event‐driven pharmacology exhibited a direct degradation of the protein of interest without requiring frequent dosing with subnanomolar range inhibitors. However, to achieve efficient protein degradation, an expression of ligase enzymes/ubiquitination cellular assemblies in the target cells is a prerequisite requirement. Importantly, platelets have lower expression of the E3 ligase enzymes, which prompted researchers to develop a series of Bcl‐x_L_ based PROTACs, as compiled in Figure 16 and Table 5. Therefore, substantial development of Bcl‐x_L_ PROTACs (**DT2216** *R* **‐PZ703b**, ***R*** **‐753 b**, **XZ739**, **PZ15227**, **XZ424**) has been carried out since the year 2019.

**Table 5:** A summary of the strategies implemented to improve the on‐target toxicity of Bcl‐x_L_ inhibitors.

Chemotype	Structural composition		Biology			Advantages	Limitations
**PROTACs**	Bcl‐x_L_ ligand	E3 ligase ligand	Cell line testing EC_50_=nM	Degradation activity (DC_50_=nM)	Implication	Target undruggable targets Overcome chemoresistance Continuous dosing is not required pM or nM range inhibitor not necessarily required Can work against mutations in the protein of interest Complete inhibition of targeted protein signaling can be achieved	Their dependence on the intracellular ubiquitin pathway for protein degradation makes their use trivial for G‐protein couple receptors of other transmembrane proteins. commonly show “Hooks effect” Poor cell permeability Synthesis is challenging Low systemic clearance
**DT2216**	ABT‐263	VHL	52^[a]^	63^[a]^	anticancer		
**PP5**	ABT‐263	VHL	32.1±3.7^[a]^	31.6±7.2^[a]^	anticancer		
**PZ703b**	ABT‐263	VHL	15.9±2.7^[a]^	14.3±5.1^[a]^	anticancer		
**PPC8**	ABT‐263	VHL	NA	20.3^[b]^	anticancer		
**753 b**	ABT‐263	VHL	NA	6.0^[b]^	anticancer		
**XZ424**	A1155463	CRBN	6.2±4.3^[a]^	50.0^[a]^	anticancer		
**XZ739**	ABT‐263	CRBN	10.1^[a]^	2.50^[a]^	anticancer		
**PZ15227**	ABT‐263	CRBN	130^[c]^	46.0	senolytic		
**SNIPERs**						Advantages as mentioned for PROTACs Can be used in cases where E3 ligases for PROTACs mutated or less expressed in the target cells.	Limitations as mentioned for PROTACs
**PROTAC‐4b**	ABT‐263	LCL161	75^[d]^	NA	anticancer		
**PROTAC‐8a**	ABT‐263	IAP compound 1	62^[d]^	NA	anticancer		
**Prodrug Strategies**						Reduces the off‐target as well as on‐target toxicity Sustained release Define release kinetics Localized delivery	Poor drug penetration Higher drug dosage
**Prodrug**	Bcl‐x_L_ ligand	Strategy		Clinical status	Implication		
**APG‐1252**	APG‐1252‐M1	Phosphate prodrugs		Phase‐I/II	anticancer		
**ABBV‐155**	NA	Antibody conjugate		Phase‐I	Anticancer		
**AZD0466**	AZD4320	dendrimer conjugate		Phase‐I	anticancer		
**Nav‐Gal**	ABT‐263	Galacto‐conjugation		NA	senolytic		

The first Bcl‐x_L_ PROTAC was **DT2216** utilized the **ABT‐263** (as Bcl‐x_L_ warhead) and VHL (as E3 ligase ligand) (as shown in Figure 6A). The information of binding conformation of **ABT‐263** with Bcl‐x_L_ was used, where morpholine ring of **ABT‐263** was found in the solvent‐exposed region and exploited as a point for a linker attachment. Therefore, the morpholine ring of **ABT‐263** was replaced with a piperazine ring (which is a bioisostere of morpholine ring) that was tethered to a VHL E3 ligase ligand. The **DT2216** showed a four‐fold potency in MOLT‐4 (EC_50_=52 nM) cells than **ABT‐263** (EC_50_=191 nM) with no platelet cytotoxicity up to 3 μM. Interestingly, **DT2216** showed a higher potency for Bcl‐2 protein than Bcl‐x_L_, while NanoBRET assays only found a stable ternary complex formation with Bcl‐x_L_, but not with Bcl‐2 protein. To enhance the Bcl‐x_L_/Bcl‐2 dual protein degradation of **DT2216**, researchers altered the linker attachment point from the morpholine ring to the methyl group of cyclohexene of **ABT‐263**. This modification led to a new class of PROTACs, where **PP5** was found more potent than its parent PROTAC (**DT2216**). **PP5** was a racemic mixture whose resolved stereochemistry led to the identification of a highly potent and cell‐selective *R*‐epimer (**PZ703b**), as shown in Figure 7A. Keeping the same warheads (**ABT‐263** and VHL E3 ligase ligand) with a subtle change in the rigidity of the linker led to the identification of dual Bcl‐x_L_/Bcl‐2 degrader (**PPC8**). The stereochemical resolution of **PPC8** led to the separation of *R*‐epimer (***R*** **‐753 b**), a highly potent and cell‐selectivity than its other counter *S*‐epimer (as shown in Figure 7B). To expand the scope of Bcl‐x_L_ PROTACs, four new series were developed (as shown in Figure 10) where various linker‐types, the attachment points on **ABT‐263** and E3 ligase ligands, were studied. Most potent PROTACs from series (Series‐1: **DT2216**, **PROTAC‐2 b**; Series‐2: **PROTAC‐6 c**; Series‐3: **PROTAC‐12 c**; Series‐4: **PROTAC‐15 a**, **PROTAC‐16 a**, **XZ739**) were tested for their cell‐selectivity against cancer cell lines (MOLT‐4, RS4;11, and H146) and platelets, as compiled in Table 3. Based on the design concept used for the development of series‐3, **PZ15227** was discovered as a Bcl‐x_L_‐based senolytic agent with reasonable cell‐selectivity compared to the **ABT‐263**. Interestingly, **ABT‐263** was the only Bcl‐x_L_ warhead utilized in all the reported PROTACs except **XZ424** which used another Bcl‐x_L_ warhead (**A1155463**). In cellular studies, **XZ424** showed 120‐fold less platelet toxicity than its parent (**A1155463**).

As reports have shown a susceptibility of E3‐ligases to the mutations and lower expression in some target cells, other approaches of protein degradation were also developed. This led to a replacement of the E3 ligase ligand with an IAP protein inhibitor. IAP proteins are the negative regulator of cellular apoptosis and consist of the ubiquitin‐associated domain. These PROTACs, which are based on IAP protein inhibition, are called SNIPERs (*S*pecific and *n*on‐genetic *I*AP‐dependent *p*rotein *e*rasers). A synthetic strategy similar to **DT‐2216** was employed to attain **PROTAC‐4 b** and **PROTAC‐8 a** (as shown in Figure 12) by replacing the morpholine ring of **ABT‐263** with piperazine and tethering it with an XIAP antagonist **(LCL161**) and **IAP compound 1**, respectively. **PROTAC‐8 a** showed more than 1000‐folds (IC_50_=8500 nM in platelets) cancer cell‐selectivity (MyLa1929, IC_50_=62 nM) compared to the moderate cell‐selectivity (MyLa1929, IC_50_=50 nM; platelet IC_50_=189 nm) of **ABT‐263**. The SAR studies indicated the specificity of IAP ligands is critical for degradation activity, as SNIPERs with **LCL161** showed moderate‐to‐low MyLa1929 activity than the SNIPERs derived from **IAP compound 1**.

Other than protein degradation strategies (PROTAC and SNIPER), researchers also applied prodrug approaches to reduce the on‐target platelet toxicity of Bcl‐x_L_ inhibitors. A phosphate prodrug‐based Bcl‐x_L_ inhibitor (**APG‐1252**) showed relatively lower cell‐permeability for platelets than cancer cells and hydrolyzed intracellularly into its active form, **APG‐1252‐M1** (also named as **BM‐1252‐M1**) (as shown in Figure 13). In animal cancer models, prodrug form (**APG‐1252**) showed a 30‐folds less platelet cytotoxicity than active‐form (**APG‐1252‐M1**).^[49]^ While another prodrug based on antibody‐Bcl‐x_L_ inhibitor conjugate (**ABBV‐155**) is in the phase‐I clinical trial. AstraZeneca developed a dendrimer (**AZD0466**) of a Bcl‐x_L_/Bcl‐2 dual inhibitor (**AZD4320**), which showed improved metabolic stability and a reversible dose‐dependent decrease in platelet count. While a galacto‐conjugate prodrug of **ABT‐263** (**Nav‐Gal**) easily gets hydrolyzed by lysosomal *β*‐galactosidase (SA‐β‐gal) in senescent cells, which produces significant cytotoxicity to senescence cells compared to the platelets.

As exemplified from the examples of PROTACs and SNIPERs in this manuscript, a reasonable cell selectivity over platelet toxicity was successfully achieved. However, there are certain underlying issues, such as inadequate aqueous solubility, unavailability of biocarriers for cellular update, and poor systemic clearance, which limit their wider applicability and implementation. However, the pharmacokinetic issues are mainly related to their larger molecular size, higher topological polar surface area, and high lipophilicity. Some attempts were made to incorporate the photopharmacology elements to these PROTACs to address these issues. Photocage and photoswitchable PROTACs are prime examples of such photopharmacology. Photoswitchable PROTACs, not only provide the spatiotemporal control over activation and inactivation of the PROTACs but can also assist in improving their cellular specificity and systemic clearance. In conclusion, approaches such as PROTACs and SNIPERs utilize event‐driven pharmacology, whereas prodrug strategies that are discussed in the paper utilize occupancy‐driven pharmacology, have shown promising results, which are compiled as shown in Table 5.

## Conflict of interest

The authors declare no conflict of interest.

## Biographical Information

*Arvind Negi obtained his Ph.D. (2019) from the School of Chemistry, National University of Ireland Galway (Ireland). He was employed at KelAda Pharmachem (Dublin, Ireland) and seconded on EU Horizon‐2020 project to Departamento de Química Orgánica (Universidad de Córdoba, Spain) for one year. In early 2021, he joined the DIT University, India as Assistant Professor (Medicinal Chemistry). After one semester, he moved to the Department of Bioproducts and Biosystems, Aalto University (Finland)*.

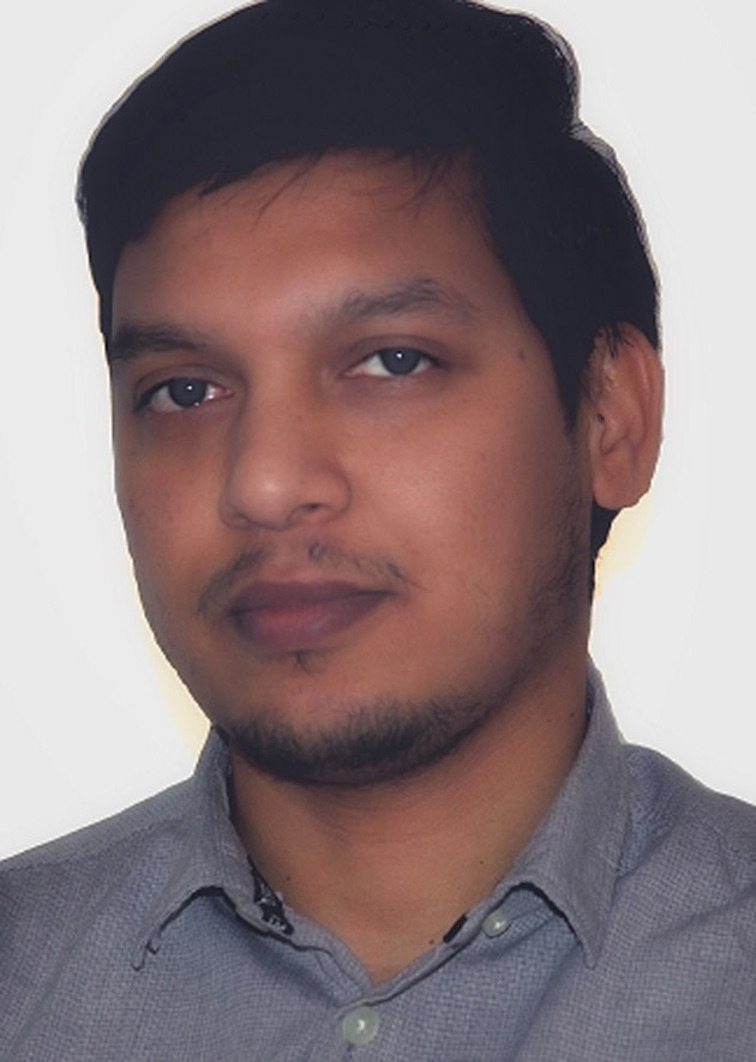

## Biographical Information

*Anne Sophie Voisin‐Chiret is a professor of medicinal chemistry at the Faculty of Pharmacy, University of Caen Normandy (CERMN) since 2015. Her research interests include medicinal chemistry in the field of protein‐protein interactions to design oligo(het)aromatic compounds that mimic proteins to fold into well‐defined conformations, such as helices and b‐sheets. Since 2012, she has led a research studying protein‐protein interfaces to design drug‐like modulators of protein‐protein interactions, particularly in oncology and neurodegenerative diseases*.